# *Saccharomyces cerevisiae* Fermentation-Derived Postbiotics Supplementation to Dairy Calves: Effects on Growth, Metabolism, Immune Status and Preliminary First Lactation Outcomes

**DOI:** 10.3390/ani15182728

**Published:** 2025-09-18

**Authors:** Marta Sfulcini, Vincenzo Lopreiato, Fiorenzo Piccioli-Cappelli, Vania Patrone, Marta Bisaschi, Ilkyu Yoon, Alessandro Maria Zontini, Mario Barbato, Luca Cattaneo, Ivonne Archetti, Erminio Trevisi, Andrea Minuti

**Affiliations:** 1 Department of Animal Science, Food and Nutrition (DIANA), Faculty of Agricultural, Food and Environmental Sciences, Università Cattolica del Sacro Cuore, 29122 Piacenza, Italy; marta.sfulcini@unicatt.it (M.S.); fiorenzo.piccioli@unicatt.it (F.P.-C.); luca.cattaneo@unicatt.it (L.C.); erminio.trevisi@unicatt.it (E.T.); 2 Department of Veterinary Sciences, Università di Messina, 98168 Messina, Italy; vilopreiato@unime.it (V.L.); mario.barbato@unime.it (M.B.); 3 Department for Sustainable Food Process—DiSTAS, Università Cattolica del Sacro Cuore, 29122 Piacenza, Italy; vania.patrone@unicatt.it (V.P.); marta.bisaschi1@unicatt.it (M.B.); 4 Diamond V Inc., Cedar Rapids, IA 52404, USA; iyoon@diamondv.com; 5 Cargill Animal Nutrition and Health, 29017 Fiorenzuola D’Arda, Italy; alessandro_zontini@cargill.com; 6 Istituto Zooprofilattico Supramental della Lombardia e dell’Emilia-Romagna, Via A. Bianchi 9, 25124 Brescia, Italy; ivonne.archetti@izsler.it

**Keywords:** calf, metabolic profile, immune status, postbiotic

## Abstract

This study evaluated the effects of supplementing dairy calves with *Saccharomyces cerevisiae* fermentation-derived postbiotics (SCFP) on growth, metabolism, immune status, and first lactation performance. Eighteen Holstein heifer calves were blocked by birth body weight and serum total protein and randomly assigned to control (CTR; *n* = 9; no supplementation) or SCFP (*n* = 9; 1 g/d SmartCare^®^ in milk replacer until weaning plus 5 g/d NutriTek^®^ until 70 d; Diamond V™, USA). Calves were weaned at 60 d and monitored until 160 d. Feed intake did not differ between groups. SCFP calves had greater post-weaning average daily gain from 71 to 100 d (0.93 vs. 0.60 kg/d, SCFP and CTR, respectively) and body weight from 100 to 160 d. They tended to have greater plasma β-hydroxybutyrate at 60 (0.32 vs. 0.27 mmol/L, SCFP and CTR, respectively) and 70 d (0.46 vs. 0.42, SCFP and CTR, respectively) and urea at 70 d (4.89 vs. 4.33 mmol/L, SCFP and CTR, respectively) and had greater acetate (515 vs. 384 μmol/L, SCFP and CTR, respectively) and propionate (33.13 vs. 22.4 ± 4.86 μmol/L, SCFP and CTR, respectively) at 60 d. SCFP calves also had lower nonesterified fatty acids at 21 d (0.23 vs. 0.38 mmol/L, SCFP and CTR, respectively), suggesting reduced energy mobilization during the most critical pre-weaning stage. Plasma myeloperoxidase was greater at 70 d (340 vs. 262 U/L, SCFP and CTR, respectively), as was phagocytosis by polymorphonuclear neutrophils at 60 (+10.4%) and 70 d (+8.2%). Feeding SCFP increased rumen activity and plasma volatile fatty acid concentrations, likely due to enhanced nutrient absorption and reduced weaning stress. SCFP calves exhibited a better immune response to lipopolysaccharide stimulation, as indicated by leukocyte gene expression, MPO, and PMN phagocytosis. Metagenomic analyses showed minor but significant changes in early-life microbiota composition at 7, 21, and 42 d. During first lactation, SCFP cows produced 2.1 kg/d more milk in the first 100 days in milk compared with CTR. In conclusion, early supplementation with SCFP supported rumen development, improved metabolic and immune function, and may enhance future productivity in dairy cows.

## Simple Summary

This study evaluated the effects of *Saccharomyces cerevisiae* fermentation-derived postbiotics (SCFP) on dairy calves’ growth, metabolism, immune function, gut microbiota and first lactation performance. Calves receiving the supplement showed improved growth after weaning, better rumen development, and a stronger immune response. They also reached greater body weight at calving and produced more milk with greater fat and protein contents in early lactation. Overall, early-life postbiotics supplementation supported healthier growth and long-term milk yield.

## 1. Introduction

The goal of calf rearing is to obtain healthy animals with greater growth rates that can become high-producing cows in the future [1]. Improved management and nutritional strategies significantly enhance the growth, feed efficiency, and overall health of young calves. Conversely, poor growth rates can lead to underweight calves at weaning, which hampers their post-weaning development and cannot be fully corrected through later nutrition [2]. In neonatal ruminants, the rumen is relatively underdeveloped because the young animal primarily relies on the abomasum and intestine for digestion [3]. When young ruminants consume solid food, it promotes microbial fermentation and VFA production, as well as physical stimulation and the function of the rumen papillae leading to development of the forestomach. The optimal development of the rumen and its papillae is essential for a healthy weaning phase [4]. The transition from a liquid to solid diet must not be a source of stress for the animal in that it negatively impacts growth and increases the risk of contracting diseases.

Newborn calves are immunologically naïve at birth [5,6]. The ingestion of colostrum is essential for providing neonates with immunologic protection during the first weeks of life [7]. Managing calves’ health is critical to minimize the risk of disease and inflammation by using appropriate nutrition, hygiene, and disease prevention strategies to reduce the risk of infections. If not properly managed the energy that should contribute to growth is used to support addressing disease or inflammation by the immune system to defend against the pathogens or support immune functions [8].

A possible ally to improve calf performance and immune system function is the use of postbiotics. *Saccharomyces cerevisiae* have been utilized for a long time as a component in domestic animal diets [9]. *Saccharomyces cerevisiae* fermentation-derived postbiotics (SCFP) are feed additives consisting of functional metabolites and bioactive compounds produced through a controlled fermentation process, and they have been included in animal diets [10]. The functional metabolites include the cell wall of yeast, composed of oligosaccharides, such as glucans and mannans [11]. In addition, the anaerobic fermentation of the yeast generates various compounds, such as B vitamins, antioxidants, phytosterols, short-chain fatty acids, and organic acids, among others [12].

In adult ruminants, SCFP have been found to improve nutrient utilization, modify rumen fermentation, and enhance production parameters [13] as well as affect the immune system. For example, in dairy cows, the feeding of SCFP during the transition period induced inflammatory biomarkers, potentially priming the immune system [14] and altering antioxidant capacity and inflammation in the postpartum period [15]. In newborn calves, the use of SCFP was shown to influence DMI, rumen pH, and nutrient digestibility and enhance the growth of rumen cellulolytic bacteria [16]. This boost in microbial activity can facilitate the shift from a liquid to a solid diet in young ruminants [4], and the use of SCFP improved growth in calves [17].

Some SCFP are water-dispersible and can be more easily supplemented to calves in their milk replacer. In this way, the postbiotic is delivered to the abomasum through the reticular groove and can contribute to stabilizing the gut microbiota and reducing the risk of pathogen colonization in the small intestine, which can cause infection and diarrhea [18]. Other preparation and distribution methods of postbiotics are delivered through the calf starter and, in this case, the effects of the product are mostly directed to the rumen. To the best of our knowledge, no studies have investigated the long-term effects (post-weaning phase and first lactation performance) of supplementing these products during the early stages of life. Most existing studies have focused on short-term outcomes, highlighting the need for further research to assess the lasting impacts of early supplementation on future production.

It was hypothesized that supplementing calf diets with *Saccharomyces cerevisiae* fermentation-derived postbiotic (SCFP) could enhance rumen and immune system functions, potentially leading to improved metabolism, and overall health. Hence, the primary aim of this study was to evaluate the effects of supplementing *Saccharomyces cerevisiae* fermentation-derived postbiotic (SCFP) from 3 to 70 days of age on the performance, metabolism, and immune status of calves. Furthermore, the opportunity arose to monitor the production performance of these animals during their first lactation.

## 2. Materials and Methods

### 2.1. Animals Management and Experimental Design

The research was conducted in accordance with Italian laws on animal experimentation and ethics, under the authorization of the Italian Ministry of Health (Authorization No. 130/2022-PR in agreement with D. Lgs.no. 26, 4 March 2014). The study was performed at Università Cattolica del Sacro Cuore dairy farm (Cerzoo, San Bonico, Piacenza, Italy). From September 2021 to March 2022, a total of 18 female Holstein calves were enrolled in the study. Calves were separated from their dams within 1 h from birth and moved to individual hutches bedded with straw. Calves were cleaned, weighed, and the navel was sprayed with oxytetracycline hydrochloride (Neo Spray Caf^®^ Aerosol; Gellini S.p.a., Aprilia, Italy). Within the first 3 h of life, calves received 3 L of colostrum. Colostrum was previously collected from several cows, pooled to obtain a brix value of 27%, and subsequently frozen in aliquots of 3 L each. If the calf did not voluntarily consume the entire 3 L, an esophageal feeder was used to deliver the remaining volume of colostrum (Speedy Drencher XL, Agri-Zoo San Marino srl, Domagnano, San Marino). The second and third meals were transition milk and offered at 12 and 24 h of life, respectively. The transition milk was pooled from the 2nd and 3rd milkings of several cows before the beginning of the study and frozen in 3 L aliquots in order to feed the same quality of transition milk to all calves.

At 3 d of age, calves were allocated using a blocked randomization based on birth weight (BW) and total serum protein (STP) at 24 h after birth, to ensure comparable baseline characteristics. Calves were allocated to either the control (CTR; *n* = 9; no supplementation; BW = 39.50 ± 4.07 kg) or supplemented with 1 g/d of SmartCare^®^ (Diamond V™, Cedar Rapids, IA, USA) in the milk replacer until weaning along with 5 g/d of NutriTek^®^ (Diamond V™) until 70 d old (SCFP; *n* = 9; BW: 39.56 ± 3.47 kg). The protein concentrations in blood serum 24 h after colostrum intake were on average 6.77 ± 0.63 g/dL for CTR and 6.81 ± 0.68 g/dL for SCFP. The SCFP group received 1 g/d of SmartCare^®^ (Diamond V™) in the milk replacer from 3 to 60 d of age along with 5 g/d of NutriTek^®^ (Diamond V™) orally dosed from 5 to 70 d of age. The calves received the treatment of NutriTek^®^ as a mixture of 5 g of grounded calf starter and 5 g of NutriTek^®^ (Diamond V™) plus 15 mL of water that was delivered by a syringe equipped with a 10 cm plastic tube. From 5 to 70 days of age, the CTR group received an oral administration of a mixture containing 10 g of ground calf starter and 15 mL of water. In both, the administration of the treatment was slowly dribbled into the corner of the mouth while the calf’s head remained in a neutral position to avoid stimulating the suckling reflex and to reduce the likelihood of reticular groove closure, ensuring delivery to the rumen.

From 3 to 60 d, calves received acidified milk replacer (MR) prepared at the rate of 150 g/L (22.5% protein, 18% fat, and 8.4% ash). The MR was composed of whey powder, refined vegetable oils (palm, coconut, rapeseed, soybean), wheat gluten, lactose, wheat flour, maltodextrin, wheat starch, tall oil fatty acids, sugar, magnesium oxide, dextrose, and 1,2-propanediol. The chemical composition of the MR is reported in Table 1. From d 3 to 6, MR was given twice a day (0800 and 1630 h) using a nipple-bottle, and from d 7, MR was offered by an individual calf feeder system (Lupetta^®^, Crema, Italy). The daily allowance of MR for the individual feeder was prepared and distributed once a day (0800 h). The feeder kept the MR temperature at approximately 33 °C throughout the day and automatically allowed the calf to drink the milk at 3 h intervals and for a maximum of 1.5 L at each meal. Calves received 6 L from d 3 to 14 and 8 L from d 15 to 50 of age. The step-down weaning started gradually from the d 51, where calves received 6 L until 53 d, 4 L from 54 to 56 d, and 2 L from 57 to 60 d of age.

From d 4 to 70, calf starter was offered ad libitum after MR feeding. The calf starter used was a commercial product prepared and pelleted (Top starter bir Purina^®^, Land O’Lakes Inc., Arden Hills, MN, USA). The chemical composition of the calf starter is reported in Table 1. As both groups were weaned (60 d of age), chopped grass hay was individually offered ad libitum 70 d of age, refilling hayracks once daily in the morning.

From d 71 to 160, calves were moved to a co-mingled pen bedded with straw and received ad libitum TMR composed of alfalfa hay, grass hay and barley straw at ratio of 33/33/33 plus 2–3 kg/d of concentrate (Cresco start calf 180^®^, Ferrero mangimi, Italy). The chemical compositions are reported in Table 2.

All of the animals were vaccinated at d 21 with Bovisil^®^ Intranasal Rsp™ live (Merck & Co., Inc., Rahway, NJ, USA) giving them 1 mL per nostril and received an anticoccidial treatment at d 70 (Baycox Multi; Elanco Italia S.p.A, Milano, Italy).

### 2.2. Health Status, Feed Intake and Body Measurements

**Figure 1:**
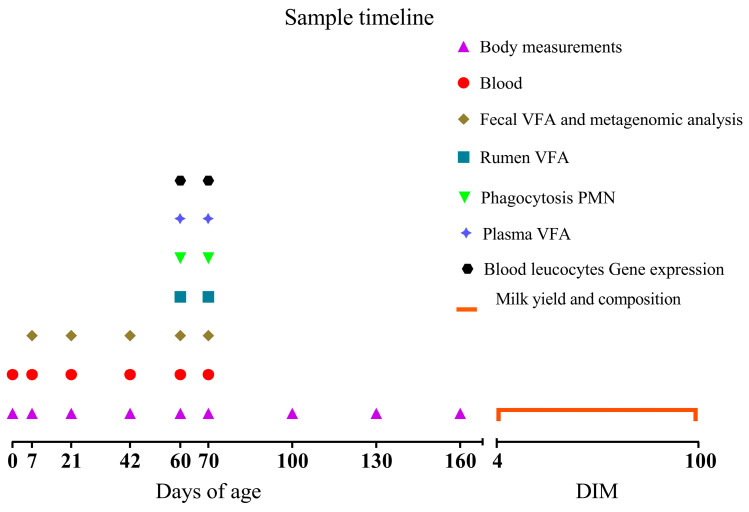
Sample timeline points of the study are reported. Calves received *Saccharomyces cerevisiae* fermentation postbiotics either 1 g/d of SmartCare (Diamond V™) in the milk replacer from 3 to 60 d along with 5 g/d of NutriTek (Diamond V™) from 3 to 70 d or no supplementation. Body measurements (body weight, wither height and heart girth) were record at 0, 7, 21, 42, 60, 70, 100, 130, 160 d of age. Blood samples were collected at 0, 7, 21, 42, 60, 70 d for the analysis of metabolic and inflammatory profile. Blood samples at 60 and 70 d were also analyzed for the concentration of plasma VFA, the percentage of phagocytosis in polymorphonuclear neutrophils (PMN) and their mean fluorescent intensity (MFI), and the blood leucocytes gene expression after the ex vivo whole blood stimulation assay with lipopolysaccharides (LPS). Fecal samples were collected at 7, 21, 42, 60, 70 d for VFA analysis and metagenomic analysis. Rumen fluid samples were collected at 60 and 70 d for VFA analysis. Milk yield, protein, fat, lactose (% and yields), and BW were recorded daily from 4 to 100 DIM.

The sample timeline is depicted in Figure 1.

The health status of calves was checked daily during the morning intake measurement by visual inspection for signs of diarrhea, respiratory disease, or other clinical disorders. None of the calves enrolled suffered from any acute health disorder during the entire study period. Body measurements of BW, wither height (WH), and heart girth (HG) were recorded at birth (d 0) and d 7, 14, 21, 28, 35, 42, 50, 60, 70, 100, 130, and 160. Body weight was obtained at each time point using an electronic scale. The measurements were recorded in the morning before feeding the MR and calf starter. The ADG was calculated utilizing the BW data from two consecutive measurements. Every morning from day 0 to 70 d of age, any remaining starter feed and MR were measured. The individual feed intake was calculated as the difference between feed offered the previous day and feed refused the following morning.

### 2.3. Blood Samples and Analysis

Blood was withdrawn from the jugular vein. Twenty four hours after colostrum administration, blood samples were collected into a clot activator tube, centrifuged to obtain serum, and the total serum protein was then measured with a handheld refractometer (PU-ATC temperature compensated, Kernco Instruments Co., El Paso, TX, USA) to assess the passive transfer of immunity from the colostrum. Blood samples were collected at 7, 21, 42, 60, and 70 d of age into lithium-heparin tubes and K-EDTA tubes (BD Vacutainer^®^, Becton, Dickinson and Company, Franklin Lakes, NJ, USA) before the morning MR meal. Lithium-heparin tubes were immediately cooled in an ice-water bath and then centrifuged at 3500× *g* for 15 min at 4 °C. Plasma was harvested, divided into aliquots, and stored at −20 °C. Plasma metabolites were analyzed at 37 °C with an automated clinical analyzer (ILAB 650; Instrumental Laboratory, Milano, Italy) as previously described [19]. Metabolites assessed were calcium (Ca), phosphorus (P), magnesium (Mg), sodium (Na), potassium (K), zinc (Zn), chlorine (Cl), glucose, total cholesterol, urea, ceruloplasmin, total protein, albumin, aspartate-aminotransferase (AST/GOT), γ-glutamyl (GGT), alkaline phosphatase (ALP), bilirubin, haptoglobin, nonesterified fatty acids (NEFA), BHB, creatinine, paraoxonase (PON), myeloperoxidase (MPO), total reactive oxygen metabolites (ROM), ferric reducing antioxidant power (FRAP), and advanced oxidation protein products (AOPP). In addition, at 60 and 70 d of age, an aliquot of plasma was stored at −80 °C for VFA analysis.

K-EDTA tubes were immediately sent to Istituto Zooprofilattico Sperimentale della Lombardia e dell’Emilia-Romagna “Bruno Umbertini” (Strada della Faggiola, 1, 29027, Gariga, PC) for assessing complete blood count (automated hematology analyzer for veterinary use XN-V-1000, Sysmex, Kobe, Japan). Parameters considered in the current study were total white blood cells (WBC) count and the WBC differential counts for neutrophils, eosinophils, basophils, lymphocytes, and monocytes.

### 2.4. Blood Leucocytes Gene Expression After Whole-Blood Ex Vivo LPS Challenge

At 60 and 70 d of age, an additional blood sample was collected into a 10 mL tube containing lithium-heparin (BD Vacutainer^®^, Becton, Dickinson and Company, Franklin Lakes, NJ, USA) to perform an ex vivo whole blood stimulation assay (WBA) with LPS according to Jahan et al. [20]. Briefly, 3 mL of whole blood was incubated for 3.5 h with LPS (*Escherichia coli* O111:B4, Sigma–Aldrich Company Ltd., Darmstadt, Germania, Cat. No. L3012) using 0.01 µg of LPS/mL of blood. After 3.5 h of incubation, blood leucocytes were collected by centrifugation (8500× *g* for 10 min at 4 °C) and isolated by washing samples with a red blood cell lysis buffer until a white blood cell pellet was obtained followed by the addition of 1 mL of TRIizol™ (Invitrogen Corp., CA, USA). Samples were stored at −80 °C. The total RNA was then extracted using the RNeasy Mini Kit (Qiagen, Hilden, Germany), and residual DNA was removed using the RNase-Free DNase Set (Qiagen, Hilden, Germany), following the manufacturer’s protocols. After extraction, RNA was quantified using the Qubit™ RNA BR Assay Kit (Thermo Fisher Scientific, Waltham, MA, USA) and RNA quality was assessed using the Experion™ Automated Electrophoresis System (Bio-Rad, Hercules, CA, USA). The average of the RNA quality index for the 36 samples was 9.1 (range: 6.5–9.6). Synthesis of cDNA was performed using a reverse transcription kit (RevertAid RT Reverse Transcription Kit; Thermo Fisher Scientific, Monza, Italy), as previously described. The resultant cDNA was diluted [1:4 (vol/vol) with DNase/RNase-free water] and then stored, at −80 °C before quantitative PCR (qPCR) testing. The qPCR procedure was performed according to Florida et al. [21] using an Optical 384-Well Reaction Plate (CFX384 Touch; Bio-Rad, Hercules, CA, USA). The qPCR efficiency and quantification cycle values were obtained for each reaction using LinRegPCR (Version 2017.1; Amsterdam UMC, Amsterdam, The Netherlands). The analyzed genes included four internal control genes (*SDHA*, *GAPDH*, *RPL13A*, and *TBP*). Genes selected for transcript analysis were those related to recognition and immune mediation functions *(CD14*, *CD36*, *TICAM1*, *TLR2*, and *TLR4*), migration and cell adhesion (*CCR2*, *CX3CR1*, *ITGB2*, *ITGAL*, *SELL* and *SELPLG*), antimicrobial strategies (*MMP9*, *LYZ*, *MPO*, *LTF* and *LCN2*), oxidative stress (*SOD1*, *SOD2* and *NOS2*) and those related to the inflammatory cascade *(NLRP3*, *IRAK1*, *IL1B*, *IL1R*, *IL8* and *IL4*). Additional details regarding sequence and amplicon size of primers used to examine gene expression are included in the Supplementary File (Table S1)

### 2.5. Phagocytosis Capacity of Polymorphonuclear Neutrophils (PMN)

At 60 and 70 d of age, a blood sample was collected into a 10 mL tube containing lithium-heparin (BD Vacutainer^®^, Becton, Dickinson and Company, Franklin Lakes, NJ, USA) to assess the phagocytic capacity of the PMN according to the method described by Hulbert et al. [22]. Briefly, two whole blood aliquots of 200 μL were transferred into 2 tubes: one as a negative control and one containing 80 μL of fluorescently labeled *E. coli*. The tubes were incubated at 38.5 °C for 10 min and then cooled in an iced bath for 10 min. The erythrocytes were hypotonically lysed adding 800 μL of ice cold Milli-Q^®^ PF water followed by the quick addition of 200 μL of 5× Phosphate-Buffered Saline (PBS) to avoid excessive damage of cells. The tubes were centrifuged at 990× *g* for 5 min at 4 °C and the supernatant was discarded. The lysing process was repeated twice. The cells were then washed using 2 mL of PBS 1× and centrifuged at 990× *g* for 5 min at 4 °C. Cells were resuspended with 200 μL of PBS 1× and analyzed with flow cytometry (BD Accuri™ C6 Plus Personal Flow Cytometer, Becton Dickinson Biosciences, San Jose, CA, USA). Data were analyzed according to Scatà et al. [23] using flow-cytometer analysis software (BD Accuri™ C6 Plus Software) to obtain the percentages of phagocytic cells in the polymorphonuclear population (phagocytic activity) and their mean fluorescent intensity (MFI).

### 2.6. Fecal and Rumen Samples

At 7, 21, 42, 60 and 70 d of age, fecal swabs were collected using a cytobrush passing through a tube of 1.1 cm of diameter inserted into the rectum. The samples were collected in duplicate. Each cytobrush collected approximately 1 g of fecal material that was then stored in a 2 mL tube at −20 °C for VFA and microbiome analysis.

At 60 and 70 d of age, rumen fluid (50 mL) was collected by esophageal tube and a vacuum pump 4 h after MR meal and calf starter delivery. The pH was measured immediately (GLP 21, Crison Instruments, SA, Alella, Barcelona, Spain). The samples were stored in aliquots of 1 mL and frozen at −20 °C until VFA, ammonia, and lactate analyses.

### 2.7. Gas Chromatography for VFA Analysis

For fecal samples, 1 mL of distilled water was added to the samples, followed by 1 min of stirring. The mixture was then centrifuged at 3000× *g* for 10 min at 4 °C and the supernatant was harvested for analysis. The rumen samples were thawed in warm water (37 °C) and centrifuged at 10,000× *g* for 10 min at 4 °C. One aliquot of rumen supernatant sample was analyzed for ammonia, L- and D-lactate with an automated clinical analyzer (ILAB 650; Instrumental Laboratory). The supernatant of rumen and fecal samples was transferred to a new tube and mixed with oxalic acid (0.12 M) and pivalic acid (0.1%), serving as an internal standard, in a proportion of 2:1:1 for the sample, oxalic acid, and pivalic acid, respectively. The mixture was vortexed and centrifuged at 10,000× *g* for 10 min. The resulting supernatant was transferred to a gas chromatography vial.

For the plasma samples, the plasma was thawed in warm water. A volume of 200 µL of plasma was transferred into a 1.5 mL tube, to which 100 µL of pivalic acid (0.005%) and 20 µL of Carrez I solution were added, followed by vortexing. Then, 20 µL of Carrez II solution was added, and the mixture was vortexed again. Finally, 20 µL of 25% metaphosphoric acid was added, and the mixture was vortexed once more. The sample was centrifuged at 10,000× *g* for 10 min at 4 °C, and the supernatant was transferred to a gas chromatography vial. The concentration of VFA was analyzed by gas chromatography using a gas chromatograph (model 7820A; Agilent Technologies, Santa Clara, CA, USA) equipped with a capillary column (30 m × 250 μm × 0.25 μm; DB-FFAP, Agilent J&W GC column) and a flame ionization detector according to [24]. Additional details are included in the Supplementary File. Data from the rumen and fecal samples were expressed as concentration for the total amount of VFA and as the molar percentage of each VFA relative to the total VFA. The ratios of acetic acid to propionic acid (C2/C3) and the sum of acetic acid and propionic acid to butyric acid ((C2 + C3)/C4) were calculated. For plasma, the total amount of VFA and the individual VFA were expressed as concentrations.

### 2.8. Fecal DNA Extraction, 16S rRNA Gene Amplification, and Illumina Sequencing

The metagenomic analysis of gut microbiota was conducted by extracting DNA from 500 mg of fecal samples with FastDNA™ SPIN Kit for Soil (MP Biomedicals, Irvine, CA, USA), according to manufacturer’s instructions with the exception of the mechanical bead beating with the FastPrep machine in which two cycles of 50 s at 6.5 m/s each were performed [25]. DNA quantity was measured with Qubit^®^ dsDNA HS Assay Kit (Life Technologies, Carlsbad, CA, USA), and DNA integrity verified with 1% agarose gel electrophoresis.

The V3-V4 sections of the bacterial 16S rRNA gene were the target of DNA amplifications using the primers 343F (5′-TACGGRAGGCAGCAG-3′) and 802R (5′-TACNVGGGTWTCTAATCC-3′). To assign sequences to samples during bioinformatics analysis, a particular seven-base tag was attached to the forward primer. The PCR protocol comprised an initial denaturation at 95 °C for three min, followed by 23 cycles of denaturation at 94 °C for 30 s, annealing at 52 °C for 30 s, and extension at 72 °C for 30 s, with a final extension at 72 °C for seven min. A 25 μL combination containing 1 μL DNA, 0.5 μM of each forward and reverse primer, and 1× KAPA HiFI hot start ready mix 2× (Kapa Biosystems, Wilmington, MA, USA) was used for each amplification reaction. After amplification, the PCR products were analyzed via agarose gel electrophoresis and quantified utilizing the Qubit HS dsDNA fluorescent assay (Life Technologies, Carlsbad, CA, USA). Amplicons were combined in equimolar concentrations and purified using the Agencourt AMPure XP PCR1 Purification system (Beckman Coulter, Brea, CA, USA). Sequencing was performed at FASTERIS SA (Plan-les-Ouates, Switzerland) using Illumina’s MiSeq platform with 300 bp paired-end mode and v3 chemistry. Following the quality checking of the raw data with FastQC v0.11.2, the bioinformatic analysis was conducted as explained by Patrone et al. [25]. Taxonomic profiling was performed using Microbiome Analyst software (version 2.0) [26].

### 2.9. Management of Heifers in Their Post-Experiment and First Lactation

After 160 d of age, the heifers were managed identically following the standard farm routine. On d 160, they were moved to another barn and received the ad libitum diet shown in Table 2. The heifers were genotyped by nasal swab at approximately 1 year of age by the 7 Genomic merit and genomic productivity, functionality and type index were similar between groups (GPFT was 3771 ± 261 vs. 3814 ± 439 and genomic transmitting ability for milk yield was 820 ± 525 vs. 783 ± 520 for CTR and SCFP, respectively). At 12 months of age, heifers were fitted with a collar containing a heat detection device using the Hr-LD tags (SCR by Allflex, Netanya, Israel). Insemination procedures were performed based on natural estrus starting at approximately 400 days of age. The 14 animals that became pregnant and subsequently entered lactation required an average of 1.4 inseminations per pregnancy (1.3 vs. 1.4 for CTR and SCFP, respectively). The age at first insemination was 426 days for the CTR group and 427 days for the treated group, while the age at conception was 434 days for the control and 444 days for the treated group. Out of the 18 calves enrolled, 14 successfully calved and entered their 1st lactation (*n* = 7 cows/group). Of the 4 missing animals, one SCFP heifer died due to trauma, and 3 were culled due to infertility (two CTR and one SCFP; not pregnant after the third AI). Two months before the expected calving, pregnant heifers were moved to the dry pen and received a TMR diet (Table 2). The average age at calving was similar between groups (24.1 ± 1 mo vs. 23.6 ± 1.7 mo for SCFP and CTR, respectively). Heifers calved and completed their first 100 days of lactation between 1 September 2023 and 25 April 2024 (temperature-humidity index = 53.3 and 49.2 in CTR and SCFP, respectively; *p* > 0.05). Upon calving, all lactating cows were moved to a freestall lactation primiparous pen together with the other primiparous cows, fed a common TMR (Table 2), and milked twice a day. Diets were formulated according to NRC [27] guidelines. Records of milk yield, protein, fat, lactose (% and yields) and milk conductibility were recorded automatically from 4 to 100 DIM using Afimilk meters and AfiLab milk analyzers (Afikim Ltd., Kibbutz Afikim, Israel) at each milking. Body weight was recorded after each milking with a single walk-in scale (Afiweigh^®^, SAE Afikim, Israel).

### 2.10. Statistical Analysis

A convenient sample size was chosen considering the intensive sampling and feeding protocols, the availability of female calves in the herd, and the need to limit the duration of the experiment to avoid introducing confounding factors. Additionally, power analyses and sample sizes reported in previous studies [28] justified the adequacy of the chosen sample size to achieve statistically significant results. Calves’ performance data (intake, body measurements, and average daily gain), blood parameters, and rumen fluid and cows’ first lactation performance were analyzed with repeated measures mixed models using the SAS GLIMMIX procedure (SAS Inst. Inc., Cary, NC, USA; release 9.4). The statistical model applied used the covariance structure with the lowest corrected Akaike information criterion (AICC) index between Compound Symmetry, First-Order Autoregressive, Toeplitz or Spatial Power [29], and included the fixed effects of treatment (CTR and SCFP), time as days of life and their interaction. Birth BW and STD at 24 h after birth were tested as covariates in the models and, as neither was significant, they were excluded from the final analyses. The model applied was the following:

Yijw = μ + Ti + Dj + TDij + Sw + εijw(1)

where: Yijw = dependent variable; μ = total mean; Ti = treatment effect (i = CTR and SCFP); Dj = effect of time (j = days of age from 0 to 160 d or days in milk from 4 to 100 DIM); TDij = interaction term between time and treatment; Sw = random effect of the calf; εijw = residual error.

For the fecal metagenomic data, alfa and beta diversity between SCFP and CTR at each time point were calculated at the species, genus and family level using the Shannon diversity index with an ANOVA test. Single-factor analysis was conducted with *p* values below 0.05, using EdgeR statistical test. Data were rarefied to the read count of the sample with lowest number of reads. SCFP samples were compared with CTR samples at species, genus, and family level, to determine the effect of the SCFP supplementation at each time point.

Data are reported as least squares means (LSM) ± mean standard error (SEM). Comparisons between groups with *p* values less than 0.05 were reported as statistically significant. Those with values between 0.05 and 0.1 were discussed as tendencies.

## 3. Results

### 3.1. Feed Intake and Growth Performance

**Figure 2:**
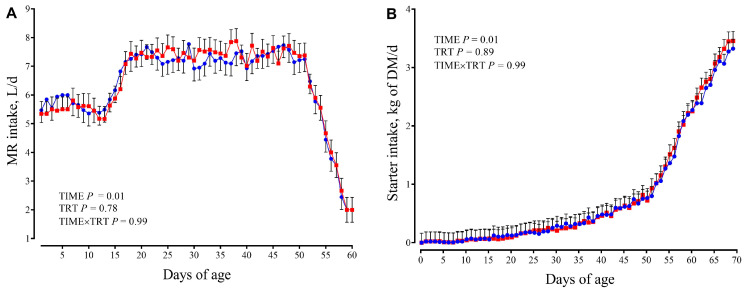
Milk replacer intake ((**A**); MR, L/d) and starter dry matter intake ((**B**); Kg/d) in calves receiving either no supplementation (CTR; *n* = 9, blue circles) or 1 g/d of SmartCare^®^ (Diamond V™) in the milk replacer from 3 to 60 d along with 5 g/d of NutriTek^®^ (Diamond V™) from 3 to 70 d (SCFP; *n* = 9, red squares). Data are presented as mean and SEM. Significance levels of the main effects of the models are reported. TRT = treatment.

Both milk and starter intake (Figure 2) did not differ between groups.

**Figure 3:**
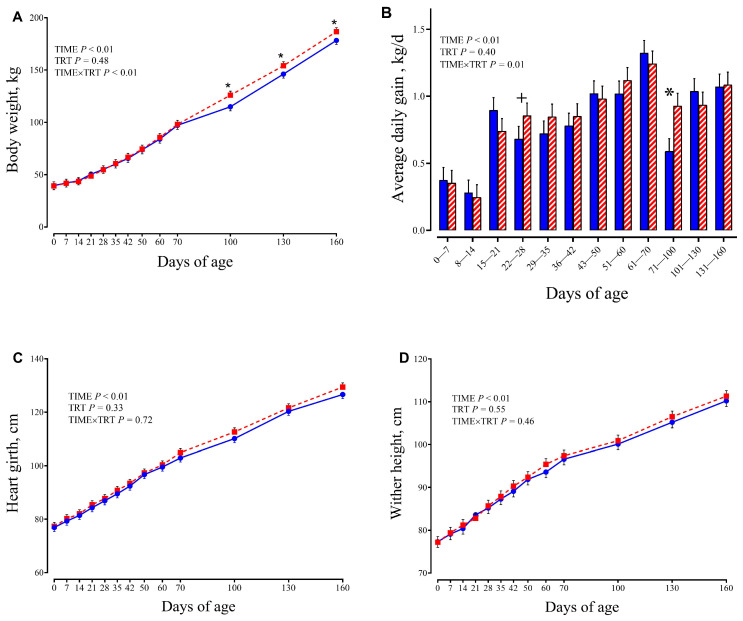
Body weight (kg; (**A**)), average daily gain (kg/d; (**B**)), heart girth (cm; (**C**)) and wither height (cm; (**D**)) in calves receiving either no supplementation (CTR, blue circles or columns; *n* = 9) or 1 g/d of SmartCare^®^ (Diamond V™) in the milk replacer from 3 to 60 d along with 5 g/d of NutriTek^®^ (Diamond V™) from 3 to 70 d (SCFP, red squares or columns; *n* = 9). Data are presented as mean and SEM. Significance levels of the main effects of the models are reported. * Indicates a significant difference (*p* ≤ 0.05), + Indicates a trend (0.05 < *p* < 0.10). TRT = treatment.

Starter intake gradually increased with age (TIME, *p* < 0.01), with mean of 2.3 kg of DM/d at 60 d (day of weaning) and 3.4 kg of DM/d at 70 d. Growth performance (BW, ADG, wither height, and heart girth) are reported in Figure 3.

Calves of both groups had similar BW at birth (38.34 ± 1.78 vs. 38.51 ± 1.78 kg, respectively for CTR and SCFP calves). However, it was different over time for each treatment (Trt × Time, *p* = 0.01). Calves had similar BW until d 70, whereas, once they were co-mingled in group pens, SCFP had greater BW than CTR calves at 100 d (126 vs. 114.9 ± 3.84 kg; *p* = 0.01, respectively), at 130 d (154.1 vs. 146.1 ± 3.84 kg, respectively; *p* = 0.04), and at 160 d (186.7 vs. 178.3 ± 3.84 kg, respectively; *p* = 0.03). The ADG was also different over time for each treatment (Trt × T, *p* = 0.01) with difference in the period 22–28 d and 71–100 d of age. The ADG from 22 to 28 d was greater in SCFP compared with the CTR group (0.86 vs. 0.68 kg/d, respectively; *p* = 0.052). From 71 to 100 d of age, SCFP showed significantly greater ADG than the CTR group (0.93 vs. 0.60 kg/d, respectively; *p* = 0.01).

### 3.2. Blood Metabolites

**Figure 4:**
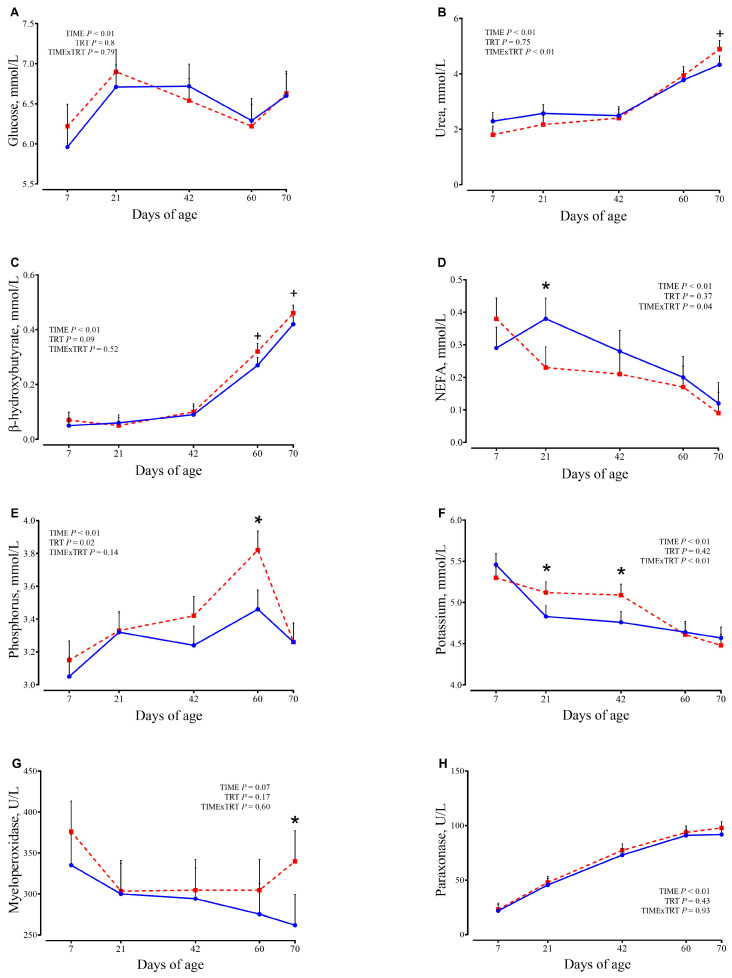
Concentrations of plasma metabolites (**A**–**H**) in calves at 7, 21, 42, 60, and 70 days of age receiving either no supplementation (CTR, blue circles and solid lines; *n* = 9) or 1 g/d of SmartCare^®^ (Diamond V™) in the milk replacer from 3 to 60 d along with 5 g/d of NutriTek^®^ (Diamond V™) from 3 to 70 d (SCFP, red squares and dashed lines; *n* = 9). Data are presented as mean and SEM. Significance levels of the main effects of the models are reported. * Indicates a significant difference (*p* ≤ 0.05), + Indicates a trend (0.05 < *p* < 0.10). TRT = treatment.

Plasma concentrations of metabolites are presented in Figure 4 and Supplementary File (Figure S1).

Total protein measured at 24 h after colostrum intake averaged 6.81 ± 0.31 g/dL for CTR and 6.85 ± 0.31 g/dL (*p* = 0.09) for SCFP calves. Greater concentrations of urea were observed for SCPF group compared with the CTR group at 70 d of age (Trt×T, *p =* 0.01; 4.89 vs. 4.33 ± 0.32 mmol/L, respectively; *p* = 0.08). Overall, phosphorus was greater in SCFP compared with the CTR group (TRT, *p* = 0.02). Potassium concentration was affected (Trt × T, *p* < 0.01) with greater concentrations in SCFP than the CTR group at 21 d (5.12 vs. 4.83 ± 0.13 mmol/L, respectively; *p* = 0.03) and at 42 d (5.09 vs. 4.76 ± 0.13 mmol/L, respectively; *p* = 0.02). The concentration of NEFA (Trt × T, *p* = 0.04) at 21 d was reduced in the SCFP compared to the CTR group (0.23 vs. 0.38 ± 0.06 mmol/L, respectively; *p* = 0.02). This trend continued until the end of the experimental period, even though no significances were detected after 21 d of age. A tendency was detected for BHB, in which SCFP calves tended to have greater concentrations than CTR calves (TRT, *p* = 0.09), mainly due to the greater concentrations at 60 d (0.32 vs. 0.27 ± 0.03 mmol/L, SCFP and CTR, respectively; *p* = 0.06) and 70 d of age (0.46 vs. 0.42 ± 0.03 mmol/L, SCFP and CTR, respectively; *p* = 0.1). The activity of MPO was greater in the SCFP compared with CTR calves at 70 d of age (339.84 vs. 262.18 ± 37.35 U/L, respectively; *p* = 0.04). The significance of the treatment for MPO was not reached. At day 7, the value of GGT tended to be reduced for SCFP calves (194.07 vs. 265.27 ± 36.96 U/L, SCFP and CTR, respectively; *p* = 0.06). All other plasma biomarkers, excluding haptoglobin and FRAP, only exhibited changes over time (*p* < 0.01). In general, albumin, paraxonase, and GOT increased, while others, such as bilirubin, alkaline phosphatase, and GGT, decreased with the increasing age of calves during the study.

### 3.3. Volatile Fatty Acids in Plasma

**Figure 5:**
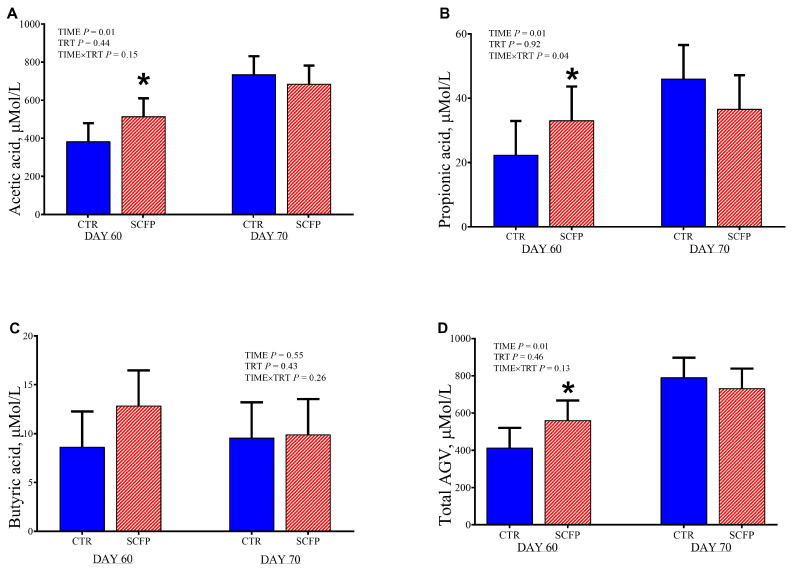
Molar proportions (**A**–**C**) of VFA and total VFA concentration (**D**) in plasma in calves at 60 and 70 days of age receiving either no supplementation (CTR, blue full columns; *n* = 9) or 1 g/d of SmartCare^®^ (Diamond V™) in the milk replacer from 3 to 60 d along with 5 g/d of NutriTek^®^ (Diamond V™) from 3 to 70 d (SCFP, red striped columns; *n* = 9). Data are presented as mean and SEM. Significance levels of the main effects of the models are reported. * Indicates a significant difference (*p* ≤ 0.05). TRT = treatment.

The concentrations of VFA in plasma are reported in Figure 5.

At 60 d of age (weaning) compared with CTR group, SCFP group had greater plasma concentration of acetic acid (515.18 vs. 384.32 ± 36.99 μmol/L, respectively for SCFP and CTR; *p* = 0.04) and propionic acid (33.13 vs. 22.4 ± 4.86 μmol/L, respectively for SCFP and CTR; *p* = 0.05). No differences were observed for plasma butyric acid concentration (*p* = 0.43).

### 3.4. White Blood Cell Differential Count

Supplementary Figure S2 presents the WBC differential results. The blood count analysis from both groups showed no significant treatment differences.

### 3.5. Gene Expression of Blood Leucocytes After Whole-Blood Ex Vivo LPS Challenge

**Figure 6:**
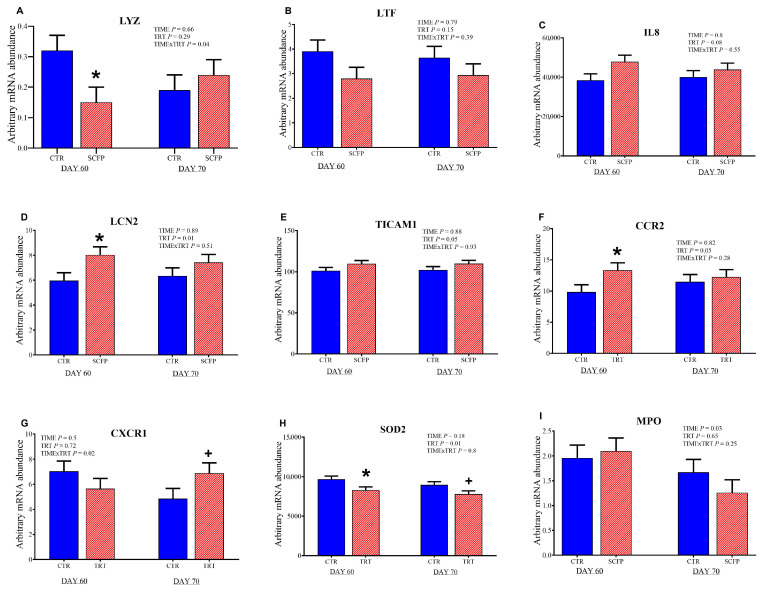
Arbitrary mRNA abundance (**A**–**I**) for gene expression in blood leucocytes after the ex vivo whole blood stimulation assay with Lipopolysaccharides (LPS) in calves at 60 and 70 days of age receiving either no supplementation (CTR, blue full columns; *n* = 9) or 1 g/d of SmartCare^®^ (Diamond V™) in the milk replacer from 3 to 60 d along with 5 g/d of NutriTek^®^ (Diamond V™) from 3 to 70 d (SCFP, red striped columns; *n* = 9). Data are presented as mean and SEM. Significance levels of the main effects of the models are reported. * Indicates a significant difference (*p* ≤ 0.05), + Indicates a trend (0.05 < *p* < 0.10). TRT = treatment.

The mRNA abundance of genes investigated in the whole blood leukocytes is shown in Figure 6 and in the Supplementary File (Supplementary Table S2).

Overall, SCFP calves had greater mRNA abundance of TIR domain containing adaptor molecule 1 (*TICAM*; TRT, *p* = 0.05), lipocalin-2 (*LCN2;* TRT, *p* = 0.01), C-C motif chemokine receptor 2 (*CCR2*; TRT, *p* = 0.05), and a tendency for interleukin 8 (*IL8*; TRT, *p* = 0.08), whereas the mRNA abundance of superoxide dismutase 2 (*SOD2*) was reduced (TRT, *p* = 0.01) compared with CTR calves. At 60 d of age, SCFP group had greater mRNA abundance of *CCR2* (*p* = 0.04) compared with the CTR group. In contrast, the CTR group had greater mRNA abundance of lysozyme (*LYZ*; *p* = 0.03) compared with the SCFP group. In the post-weaning phase, at 70 d of age, SCFP showed a tendency for lower mRNA abundance of *SOD2* (*p* = 0.09) compared with CTR.

### 3.6. Phagocytosis Capacity of PMN

**Figure 7:**
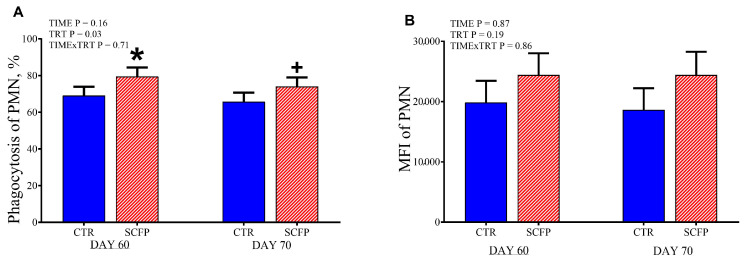
The percentage of phagocytosis in polymorphonuclear neutrophils (PMN; (**A**)) and their mean fluorescent intensity (MFI; (**B**)) after incubation with fluorescently labeled *E. coli* in calves at 60 and 70 days of age receiving either no supplementation (CTR, blue full columns; *n* = 9) or 1 g/d of SmartCare^®^ (Diamond V™) in the milk replacer from 3 to 60 d along with 5 g/d of NutriTek^®^ (Diamond V™) from 3 to 70 d (SCFP, red striped columns; *n* = 9). Data are presented as mean and SEM. Significance levels of the main effects of the models are reported. * Indicates a significant difference (*p* ≤ 0.05), + Indicates a trend (0.05 < *p* < 0.10). TRT = treatment.

PMN phagocytosis capacity data are reported in Figure 7.

Overall, the phagocytosis activity of the PMN was greater in SCFP compared with the CTR group (TRT, *p* = 0.05). The SCFP group had increased phagocytic activity of PMNs of +10.4% at 60 d (*p* = 0.04) and +8.2% at 70 of age (*p* = 0.1) compared with the CTR group.

### 3.7. Fecal and Rumen Fluid Analysis

**Figure 8:**
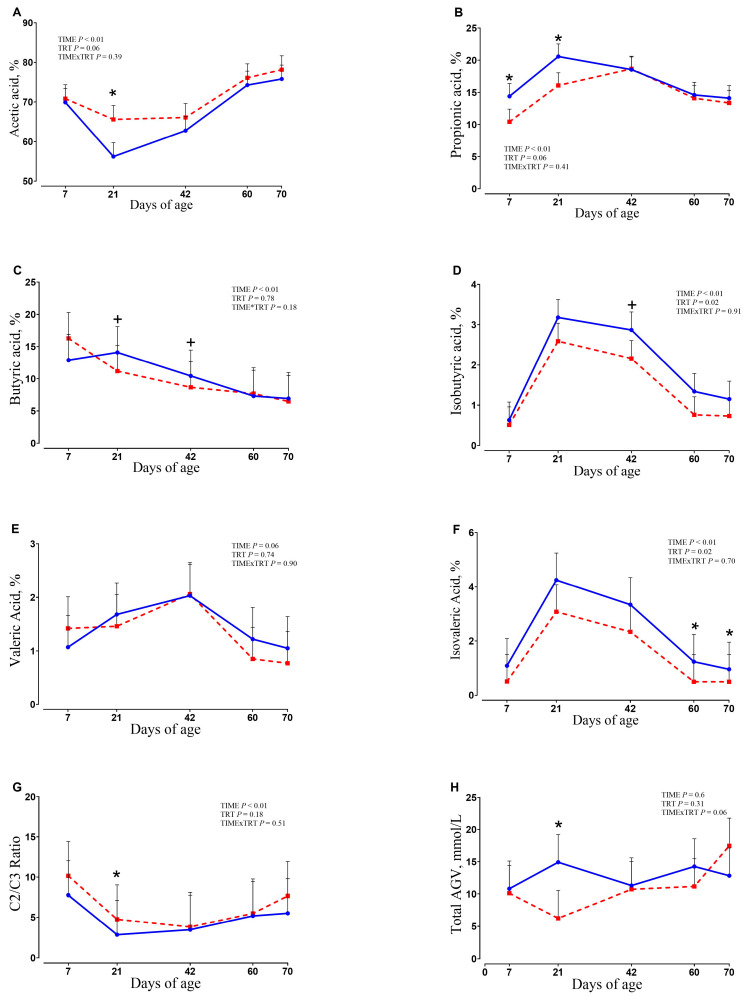
Molar proportions (**A**–**G**) of VFA and total VFA concentration (**H**) in fecal samples of calves at 7, 21, 42, 60, and 70 days of age receiving either no supplementation (CTR, blue circles and solid lines; *n* = 9) or 1 g/d of SmartCare^®^ (Diamond V™) in the milk replacer from 3 to 60 d along with 5 g/d of NutriTek^®^ (Diamond V™) from 3 to 70 d (SCFP, red squares and dashed lines; *n* = 9). Data are presented as mean and SEM. Significance levels of the main effects of the models are reported. * Indicates a significant difference (*p* ≤ 0.05), + Indicates a trend (0.05 < *p* < 0.10). TRT = treatment.

Results of total VFA and molar proportion of VFA in fecal samples are presented in Figure 8.

Overall, the proportion of acetate in fecal samples tended to be greater in the SCFP group (*p* = 0.06) compared with the CTR group, with the differences at 21 d (65.6% vs. 56.21% for SCFP and CTR, respectively; *p* = 0.01). Compared with the CTR group, the proportion of propionate was reduced in the SCFP group at 7 d (*p* = 0.05) and 21 d (*p* = 0.03). The butyrate proportion tended to be reduced in the SCFP group at 21 and 42 d of age compared to the CTR group (*p* = 0.10). The proportion of isobutyrate and isovalerate was reduced overall throughout the study in the SCFP fed calves compared with CTR (*p* = 0.02 for both). The ratio of acetate and propionate was greater in the SCFP group at 21 d of age (*p* = 0.04) compared with the CTR group. Concerning the rumen fluid samples, no significant differences were detected between treatment groups for pH, VFA, lactate, and ammonia (Figures S3 and S4).

### 3.8. Metagenomic Analysis of Fecal Microbiota

**Figure 9:**
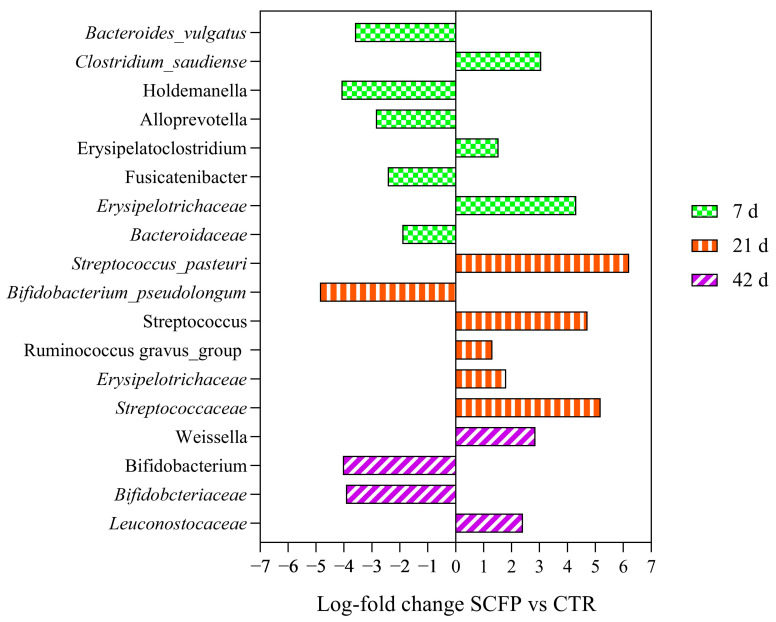
Taxa showing significant relative abundance changes (*p* ≤ 0.05) in fecal samples of calves at 7, 21 and 42 days of age in response to SCFP supplementation, as calculated using EdgeR statistical test. Pairwise comparisons were performed between SCFP group (*n* = 9; calves receiving 1 g/d of SmartCare^®^, Diamond V™, in the milk replacer from 3 to 60 d along with 5 g/d of NutriTek^®^, Diamond V™, from 3 to 70 d) and CTR group (*n* = 9; calves receiving no supplementation).

Alpha and beta diversity analysis were not significantly different between CTR and SCFP groups at any time point. Log-fold change values were calculated at each time point for all taxa with statistically significant differences in relative abundance reported in Figure 9.

The comparison between the SCFP and CTR sample groups at 7 d indicated significantly lower proportions of *Bacteroides_vulgatus*, *Holdemanella*, *Alloprevotella*, *Fusicatenibacter* and *Bacteroidaceae*, but greater *Clostridium saudiense*, *Erysipelatoclostridium* and *Erysipelotrichaceae* in the SCFP group. At 21 d, species *Streptococcus_pasteuri*, genus *Streptococcus* and family *Streptococcaceae* were significantly greater in SCFP vs. CTR calves, as well as *Erysepelotrichaceae* and *Ruminococcus_gravus_group*. Conversely, *Bifidobacterium_pseudolongum* was reduced at 21 d. At 42 d, *Weissella* and *Leuconostocaceae* were enriched in treated fecal samples, while *Bifidobacterium_psudolongum* and *Bifidobacteriaceae* were less abundant. Unlike samples at 7, 21 and 42 d, SCFP samples collected at 60 and 70 days showed no significant compositional changes when compared to CTR samples.

### 3.9. First Lactation Performance

**Figure 10:**
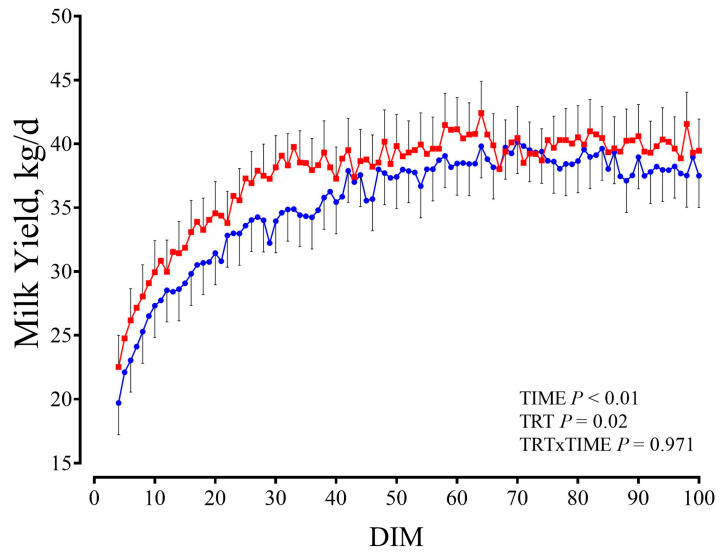
Milk yield (A) from 4 to 100 DIM of cows that from day 3 to day 70 of life received either no supplementation (CTR, blue circles; *n* = 7) or 1 g/d of SmartCare^®^ (Diamond V™) in the milk replacer from 3 to 60 d along with 5 g/d of NutriTek^®^ (Diamond V™) from 3 to 70 d (SCFP, red squares; *n* = 7). Data were recorded automatically using Afimilk meters and AfiLab milk analyzers (Afikim Ltd., Kibbutz Afikim, Israel) at each milking. TRT = treatment.

Milk yield from d 4 to 100 is shown in Figure 10.

Milk composition data are reported in Table 3.

Overall, SCFP cows had greater milk yield throughout the first 100 DIM compared with CTR cows (37.62 vs. 35.45 ± 0.65 kg/d SCFP and CTR, respectively; *p* = 0.02). Also, ECM of SCFP was increased compared with CTR cows (45.42 vs. 43.54 ± 0.65 kg/d SCFP and CTR, respectively; *p* = 0.04). Overall, milk fat percentage was reduced in SCFP cows (4 vs. 3.8 ± 0.05% SCFP and CTR, respectively, *p* < 0.01) but SCFP daily milk protein yield was greater compared with CTR cows (1.31 vs. 1.24 ± 0.02 kg/d SCFP and CTR, respectively; TRT, *p* < 0.01). BW after each milking during the first 100 DIM was not different between groups.

## 4. Discussion

The use of dietary strategies to improve calves’ performance and health is an important topic, as it can positively impact the productive performance of future dairy cows, thus enhancing the sustainability of the system. In this study, we evaluated the impact of SCFP supplementation during the early life of calves on several metabolic and immunity-related variables (0–70 d of age). Additionally, we assessed the carry-over effects on performance during the post-weaning period (70–160 d of age), and finally collected the production data of the heifers during their first lactation.

A unique aspect of this study was the method of administering the SCFP supplementation. The product was offered using two simultaneous methods in the liquid and solid feeds. For the liquid supplementation, 1 g of the product was added to the MR during the suckling period (3–60 d of age). This approach allowed the SCFP to act at the intestinal level, as the esophageal groove reflex during suckling creates a bypass of the rumen. For the solid feed, 5 g of the SCFP were administered daily from d 3 to 70. In this case, the delivery of the supplement was performed by mixing the product with milled starter feed, adding water to achieve a mush-like consistency, and administering it orally using a syringe with a short tube. Care was taken to prevent the calf from displaying sucking behavior to ensure that the product would not bypass the rumen. Previous studies use a starter feed containing the additive at a 1–2% concentration that is made available to calves ad libitum. This approach does not provide complete control over the amount of additive ingested, as it depends on the voluntary starter intake by the calf. This is a challenge, particularly in the early stages of life when starter consumption is very low.

Despite the small sample size, our results showed a significant impact on lactation performance, which suggests that early-life nutritional programming beyond nutrients can have an impact at later production stages. Furthermore, we believe that we were able to adopt a proper experimental design and sampling complexity.

### 4.1. Effect on Performance, Metabolism and Rumen Development in Calves

In our study, DMI was not different between groups. SCFP may enhance starter intake in calves; however, their effect has often been inconsistent. Our result agrees with Quigley et al. [30], and with Magalhães et al. [31], who observed no effect on DMI using SCFP supplementation, both in pre- and post-weaning periods. On the other hand, some studies using SCFP supplementation have reported greater intake before weaning [32], whereas some researchers observed positive effects on DMI only after weaning [17].

The ADG and consequently the BW showed interesting and distinct results in our study. There was no effect of SCFP during the period of supplementation (until 70 d of age), but there was a considerable and positive carry-over effect from 71 to 160 d of age due to previous SCFP supplementation. At the end of the study (160 d of age), the SCFP group was 8.4 kg heavier than the CTR group, and the period in which the SCFP group gained an advantage over the CTR group was from 71 to 100 d of age, with a 57% increase in ADG. Average daily gain is closely linked to DMI, with greater DMI typically leading to greater ADG. Since individual intake was not recorded after 70 d of age, it is difficult to ascribe this result to an increase in DMI or other factors.

Similar to the inconsistent changes in DMI observed in previous studies, ADG and BW also showed inconsistent results. Klopp et al. [33] found greater ADG only in the post-weaning period (from 57 to 112 d of age) in calves that received SCFP. Interesting results regarding the ADG have also been reported by Lesmeister [17] in a trial using an early weaning protocol, who found no differences before weaning, while in the post-weaning period, a greater ADG was observed in calves receiving 2% of DM of SCFP (XP, Diamond V™).

The inconsistency in the results previously reported may be due to the different management, product type, and timing and quantity of treatment administration. In our study, all of the treated animals received 5 g of SCFP from 5 to 70 d of age by oral dosage; however, in most studies, supplementation was conducted by adding the product to the starter. As a result, actual consumption of SCFP varies as starter intake changes. In the first weeks of life, when starter intake is limited, the actual amount of product ingested by the calf may be limited. It can be hypothesized that administering SCFP in significant quantities from the early stages of life may have a bigger impact on subsequent performance and potentially health. Early-life changes in gut microbial colonization may have permanent effects on the development of rumen microbiota and, subsequently, on the host’s phenotype [34].

In our study, a carry-over effect was evident when the calves experience the post-weaning stress of being co-mingled into group pens and transitioning to a new, less digestible ration as compared to the starter feed. Under these conditions, the improved performance observed in the SCFP group may be attributed to better rumen functionality and/or enhanced immune system condition in the calves.

In young calves, increased glucose availability is expected to enhance body weight gain. When SCFP increased DMI, plasma glucose was also positively affected [32]. Unfortunately, we did not measure either glucose or feed intake at the time when ADG was affected by the treatment, so we cannot directly confirm this mechanism. However, we hypothesize that the SCFP supplementation may stabilize feed intake and improve metabolism and mitigate post-weaning stress. Olagaray et al. [35] found a modulation of the feeding behavior in cows after the SCFP supplementation resulting in more frequent meals that may contribute to improved rumen function. Although in young calves the main source of energy are lactose and fat from milk, when the rumen begins to develop, VFAs will also contribute to energy balance. The increase in plasma β-hydroxybutyrate [17,32,36] is a result of increased ketogenesis in the rumen. Our data demonstrated a similar response in the concentration of plasma BHB quickly increased from 42 d of age at the time when starter intake increased. Quigley et al. [36] found that blood BHB increase was closely related to the availability of calf starter. Interestingly, in our study, plasma BHB was greater on d 60 and 70 in the SCFP group, but the intake of calf starter did not differ between the two groups. Our hypothesis is that the SCFP calves might have had a more active and developed rumen compared with the CTR ones, thus the difference in BHB may not solely be related to starter ingestion.

There is varying information regarding how SCFP affects the structural and functional development of the rumen [37]. Some studies suggest that supplementation of SCFP could improve rumen development, by increasing papillae length and papillae width around the time of weaning [17].

In our study, this scenario could also be confirmed by the greater concentration of plasma urea in the SCFP calves at 70 d of age. The SCFP group might have had different protein metabolism within the rumen with increased nitrogen utilization efficiency. Additionally, the concentration of VFAs in plasma supports the better rumen functionality in the SCFP group, with greater concentrations of acetic and propionic acid at 60 d of age. This result could be due to an increase in the absorptive surface of the rumen wall, which is positively correlated with the rate of VFA absorption [38]. At the same time (d 60), the plasma concentration of phosphorus was greater in the SCFP group. The SCFP may increase feed intake or dietary phosphorus absorption, or both [39]. Rumen microbiota play an important role for dietary phosphorus absorption. A significant proportion of plant-bound phosphorus is in the form of phytate, that cannot be absorbed unless being metabolized by the gut microbiota [39].

### 4.2. Effect on Health Status and Immune System in Calves

During the first few months, calves are very vulnerable to diseases from various pathogens and environmental stressors because their immune system is immature. Illnesses that reduce growth rates can lead to poor weaning weights and impaired post-weaning growth, in which future nutrition cannot fully compensate [2]. The weaning process can also be very stressful, often leading to increased respiratory issues [37]. Maintaining good health in heifers during their entire growth phase is essential to support their genetic potential during the productive phase.

In our study, calves that received the SCFP supplementation had a reduced concentration of NEFA at 21 d of age. NEFA are an important energy source. Their concentration increase in blood indicates body fat mobilization when energy intake is not enough to cover the energy requirements. Considering that the energy intake was similar between the two groups, this reduction in NEFA may indicate a greater energy need in the CTR group. Given the results reported by other studies concerning the effect of SCFP on the immune system, it can be assumed that the larger energy requirement in the CTR group is due to a greater energy requirement from the immune system [31,33]. This shift of the intake energy from growth to the immune system resulted in a reduced performance as indicated by the lower ADG from day 22 to 28 of age in the CTR group. With the decrease in passive immunity given by the colostrum, the animals’ immune system is subjected to stress. Indeed, many of the immune system components are not functional until calves are at least 2 to 4 weeks of age [40] and may continue to develop until puberty [41]. It is probable that the animals that have received SCFP have a more active immune system ready to face the challenge. Mahmoud et al. [42] found that calves treated with SCFP mounted an enhanced cytokine response compared with cells from control calves suggesting that SCFP treatment may be training or enhancing the calves systemic innate immune system. Sanchez et al. [43] found that the metabolic response to LPS challenge in crossbred beef heifers was affected by a yeast cell wall additive, with a reduced magnitude of increase in NEFA during the LPS challenge and a greater ADG in the group fed yeast cells group. The authors suggested that the dietary yeast cell wall can enhance energy metabolism during an immune challenge without causing lipolysis or muscle catabolism.

SCFP contain compounds like oligosaccharides, glucan, and mannan, which have been reported to positively affect local and systemic immune responses [12,44] and may also exhibit antimicrobial activity against pathogens [45]. These substances primarily act in the gastrointestinal tract, interacting with the microbial population that includes the naturally occurring commensal bacteria that then directly connect with the immune system.

Yeast cell walls, a component of SCFP, contain approximately 35% mannan and 30% glucan [11]; they are not digested or absorbed in the small intestine [12]. Their presence in the gut may enhance immune responses and prevent pathogen colonization, with mannan potentially blocking bacterial attachment to the intestinal epithelium. As reported by Wójcik [46], the molecular structure, type and number of side chains, tertiary structure, molecular mass, and solubility of β-glucans determine their physicochemical and immune properties. β-glucans act as antigens by stimulating specific macrophage receptors [47].

Other results supporting the positive effect on the immune system when supplementing with SCFP can be found in the plasma myeloperoxidase and the phagocytosis test. The phagocytosis capacity of the polymorphonuclear cell was significantly greater at 60 d and 70 d in the SCFP group. This agrees with Wójcik [46] that highlight increased respiratory burst activity and greater potential killing activity in calves receiving β-glucans. In addition, the myeloperoxidase at 70 d was significantly improved in the calves that received the SCFP. Myeloperoxidase is released from cytoplasmic granules of neutrophils and monocytes/macrophages by a degranulation process, reacts with the H_2_O_2_ formed by the respiratory burst to form a complex that can oxidize a large variety of substances. The primary function of neutrophils is the phagocytosis and destruction of microorganisms, and the release of MPO and H_2_O_2_ into the phagosome containing the ingested microorganism generally leads to a rapid microbicidal effect [48].

The gene expression data obtained from the ex vivo test confirmed the effect of SCFP administration on immune system functionality. Generally, a more intense response is observed in leukocytes following the inflammatory challenge with LPS. Some genes that play a crucial role in executing the immune response (*IL8*, *LCN2*, *TICAM1*, *CCR2*) showed a greater response in the SCFP group, suggesting that the immune system of these subjects is more reactive to immune challenges. Following an inflammatory stimulus, a series of mechanisms is triggered with the aim of defeating any potential pathogen. It is very important for the immune response to be strong and have a rapid resolution so that the pathogen can be eliminated with minimal repercussions for the entire organism [49]. The beneficial effects of SCFP have already been observed in other studies. In a recent study, calves receiving SCFP supplementation exhibited enhanced innate immune function, with an increased capacity of circulating peripheral blood mononuclear cell (PBMC) to secrete proinflammatory cytokines in response to LPS stimulation [42]. Interestingly, the same study found that calves receiving SCFP had reduced clinical disease, reduced lung pathology, and a reduced incidence of secondary bacterial infection following experimental respiratory syncytial virus infection, which suggested an innate training effect of SCFP.

### 4.3. Effect on Fecal Microbiota Composition

The objective of the calf feeding system is to provide the animals with optimal nourishment to enhance their growth, health, and development. An essential aspect in sustaining animal health is the composition of their gut microbiome [50,51]. In this study, fecal microbiota was examined rather than rumen because the aim was to focus on the microbiota composition at the very early stages of a calf’s life. The results of the current study showed that the alpha and beta diversity of calf fecal microbiota were not affected by the administration of SCFP. This fact had already been described by Centeno-Martinez et al. [52], who only found a statistically significant difference at 112 d. On the other hand, the present study confirmed that the supplementation of SCFP in calves may induce changes in the relative abundances of a number of specific taxa during their first few weeks of life. In fact, while SCFP supplementation induced rapid statistically significant differences from 7 d to 42 d samples, data obtained at 60 and 70 d showed no relevant changes when comparing SCFP samples with CTR samples. The same was reported by Centeno-Martinez et al. [52], where a change in the fecal microbiome composition was noticed only from 0 to 28 days, and it could be linked to an increasing stability of the gut microbiota during a calf’s growth [53]. A previous study [54] that examined the rumen microbiota, attributed the advantageous effects of SCFP on the rumen samples to the enhanced relative abundance of certain members of the *Bacteroidetes* phylum, including *Prevotella*, as well as some members of the *Ruminococcaceae*. In this study, *Bacteroidaceae* and *Alloprevotella* were reduced in fecal microbiota at 7 d, but a *Ruminococcus_gnavus_group* was increased at 21 d. In addition, lactic acid bacteria, such as Weissella and *Leuconostocaceae*, extensively known for their beneficial effect on calves in preventing diarrhea and enhanced growth, were greatly increased at 42 d [55].

Interestingly, as mentioned above, this work demonstrates that SCFP supplementation enhances VFA production in rumen, particularly with a significant increase in acetate production observed at 21 days; bacteria from the *Streptococcaceae* family are key acetate-producing microorganisms [56]. Also, the decrease in propionate at 7 and 21 d and of butyrate at 21 and 42 d may be linked to the reduction in *Bacteroides_vulgatus*, *Clostridium_saudiense* and *Bacteroidaceae* registered in feces at 7 d, since they are great producers of the latter VFAs [57,58,59].

### 4.4. Post-Experiment Performance in First Lactation

Although milk production in the first lactation was monitored from a small number of animals (*n* = 7 per treatment), the results obtained appear noteworthy. SCFP cows exhibited an improved milk yield in the first 100 DIM, which also led to a lower fat percentage and greater total protein output (kg) in milk. To our knowledge, there are no studies that explored the effects of early-life SCFP supplementation on the first lactation performance. Several studies have shown that the growth rate of heifers’ post-weaning through puberty was quadratically related to milk yield [60]. Even though these data need to be verified with larger sample sizes, they could provide an insight that the use of SCFP during the early stages of life may have positive effects on the performance of adult animals as well.

## 5. Conclusions

**Figure 11:**
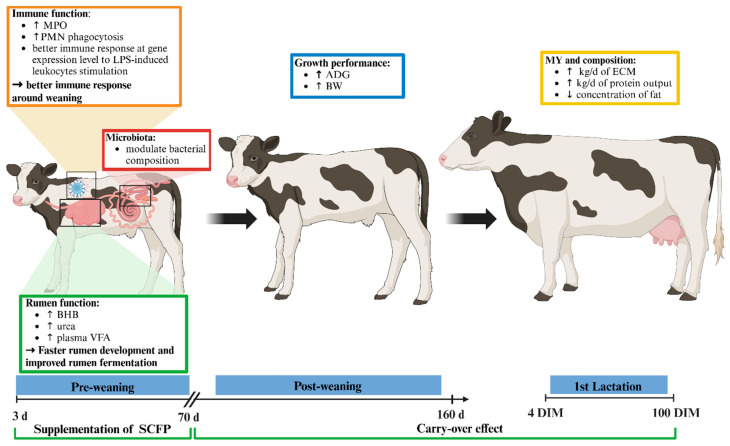
Proposed model for the effects of *Saccharomyces cerevisiae* fermentation-derived postbiotic (SCFP) supplementation in calves. SCFP supplementation during early life (1 g/d of SmartCare^®^, Diamond V™) in the milk replacer from 3 to 60 d along with 5 g/d of NutriTek^®^, Diamond V™) from 3 to 70 d positively influenced rumen development, immune function, and growth, with effects extending into the first lactation. SCFP-treated calves exhibited increased plasma concentrations of β-hydroxybutyrate (BHB), urea, and volatile fatty acids (VFA), indicating improved rumen fermentation and nutrient absorption. SCFP enhances innate immune function by increasing myeloperoxidase (MPO) plasma concentration and the phagocytic capacity of polymorphonuclear neutrophils (PMN), improving the immune response during critical periods, such as weaning. Although SCFP supplementation did not affect alpha or beta diversity of the gut microbiota, specific shifts in bacterial composition were observed, including increased acetate production, which may support gut health and energy metabolism. Treated calves also demonstrated superior growth performance during the post-weaning period, with higher average daily gain (ADG) and body weight (BW). Data on the first lactation (4–100 DIM) suggested increased energy-corrected milk (ECM) and protein production, along with reduced fat concentration. These findings indicate that early-life SCFP supplementation not only supports calf development and health but may also enhance long-term productivity. Created in https://BioRender.com.

Supplementing *Saccharomyces cerevisiae* fermentation-derived postbiotics (SCFP) to calves in the early stage of life was associated with indicators of enhanced rumen development, metabolic profile, and immune function, resulting in increased growth in the post-weaning period (Figure 11).

SCFP-supplemented calves showed greater plasma concentrations of BHB, urea, phosphorus and VFA, indicating improved rumen activity and nutrient absorption. SCFPsupplementation was also linked to greater plasma myeloperoxidase concentration and greater phagocytic activity, suggesting a more responsive immune system. Although SCFP supplementation did not influence gut microbiota alpha and beta diversity, shifts in specific bacterial taxa were observed, increasing acetate production during a calf’s early life. Despite the limited sample size, the milk production of the first 100 DIM suggest that SCFP supplementation to calves at the early stage of life may offer benefits lasting through the first lactation. Future studies with larger samples are warranted to confirm these findings and to assess their long-term relevance.

## Figures and Tables

**Table 1:** Nutrient composition (% of DM) of milk replacer (MR), calf starter, and Cresco start calf concentrate.

Item	MR ^1^	Calf Starter ^2^ Until 70 d	Concentrate ^3^ From 70 to 160 d
CP, %	22.5	16.3	18
Crude fat, %	18	3.5	2.5
Crude fiber, %	0.0	14.94	14.5
Ash, %	8.4	7.84	8
Na, %	0.71	0.39	
Ca, %	0.84		
P, %	0.64		

^1^ MR, Gold Lupetta^®^, Tredì Italia Srl, Italy. ^2^ Calf starter, Top starter bir Purina^®^, Land O’Lakes Inc., Arden Hills, MN, USA. ^3^ Concentrate, Cresco start calf 180, Ferrero mangimi, Italy.

**Table 2:** Nutrient composition (% of DM unless otherwise noted) of TMR fed during the study period.

Item	Hay Mix	TMR		
		Heifer	Heifer from −60 d to Calving	Lactation
Ingredients				
Corn silage				29.8
Concentrate mix ^1^		3.03	3.65	25.8
Alfalfa hay	34.0	25.6		26.4
Grass hay	33.0			
Barley silage		41.3	49.7	
Barley straw	33.0	15.9	29.8	2.84
Soybean meal		7.09	8.85	8.10
Sunflower meal		6.41	7.59	4.20
Hydrogenated fats				0.88
Vitamin and mineral premix ^2^		0.67	0.41	1.22
Limestone				0.36
Sodium bicarbonate				0.36
Sodium Chloride				0.04
Nutrient composition, % of DM ^3^				
Starch		9.80	11.3	27.4
CP	8.90	14.1	12.3	15.7
Metabolizable protein (MP)		8.49	8.44	10.0
Lysine (% MP)	0.32	4.7	3.8	6.57
Methionine (% MP)	0.07	1.4	1.9	2.15
NDF	60.7	49.2	52.3	31.8
ADF	38.6	31.2	32.2	19.9
Crude fat (EE)	1.28	2.59	2.65	3.61
Ash	8.30	8.13	6.60	7.44
Ca	1.18	0.64	0.31	1.21
P	0.14	0.40	0.36	0.37
Mg	0.23	0.25	0.23	0.27
K	0.58	1.31	0.86	0.78
Na	0.06	0.09	0.09	0.23
Energy, Mcal/kg of DM				
ME	1.78	2.34	1.94	2.62
NE_M_	0.92	1.53	1.28	1.79
NE_L_	0.34			1.57

^1^ Concentrate mix contained 55% corn flour, 30% barley flaked, and 15% sorghum grain meal. ^2^ Vitamin and mineral mix provided 450,000 UI of vitamin A, 45,000 IU of vitamin D, 900 mg of vitamin E, 35 mg of vitamin K, 100 mg of vitamin H1, 42 mg of vitamin B1, 0.6 mg of vitamin B12, 6000 mg of vitamin PP, 300 mg of Mn, 1030 mg of Zn, 80 mg of Cu, and 15 mg of I, 2.7 mg Se. ^3^ The DM was 89.9% for hay mix, 44.8% for heifer growth TMR, and 52.3% for lactation TMR.

**Table 3:** Average milk composition from 4 to 100 DIM of cows that from day 3 to day 70 of life received either no supplementation (CTR; *n* = 7) or 1 g/d of SmartCare^®^ (Diamond V™) in the milk replacer from 3 to 60 d along with 5 g/d of NutriTek^®^ (Diamond V™) from 3 to 70 d (SCFP; *n* = 7).

	CTR	SCFP	SEM	*p*-Value		
				TIME	TRT	TIME × TRT
Milk yield, kg/d	35.45	37.62	0.65	<0.01 *	0.02 *	0.971
Fat, %	3.97	3.78	0.05	<0.01 *	0.01 *	0.27
Fat yield kg/d	1.39	1.41	0.03	<0.01 *	0.68	0.29
Protein, %	3.49	3.49	0.01	0.2	0.94	0.8
Protein yield, kg/d	1.24	1.31	0.02	<0.01 *	0.01 *	0.77
Lactose, %	4.74	4.76	0.04	0.04 *	0.71	<0.01 *
Milk conductibility, mS/cm	8.95	9.03	0.04	0.17	0.12	0.29
ECM, kg/d	43.54	45.42	0.65	<0.01 *	0.04 *	0.81
FCM, kg/d	35.04	36.1	0.59	<0.01 *	0.21	0.41
Body weight ^1^, kg	567.99	580.17	11.95	0.90	0.48	0.75

Data are presented as mean and SEM. Significance levels of the main effects of the models are reported. * Indicates a significant difference (*p* ≤ 0.05). ^1^ Body weight was recorded after each milking with a single walk-in scale (Afiweigh^®^, SAE Afikim, Israel). TRT = treatment; ECM = energy corrected milk; FCM = fat corrected milk.

## Data Availability

The Illumina sequencing reads were deposited in the NCBI Sequence Read Archive (SRA), under the BioProject ID: PRJNA1194120 (Biosamples: SAMN45166807-SAMN45166914). [NCBI Sequence Read Archive (SRA)] [https://www.ncbi.nlm.nih.gov/bioproject/PRJNA1194120] [PRJNA1194120] accessed on 3 December 2024.

## Supplementary Materials

The following supporting information can be downloaded at: https://www.mdpi.com/article/10.3390/ani15182728/s1, protocol for gas chromatography for VFA analysis; Table S1. GenBank accession number, hybridization position, sequence, amplicon size and source of primers for Bos taurus used to analyze gene expression by qPCR; Table S2. Arbitrary mRNA abundance for gene expression in blood leucocytes after the ex vivo whole blood stimulation assay with Lipopolysaccharides (LPS) in calves at 60 and 70 days of age; Figure S1. Concentrations of plasma metabolites in calves at 7, 21, 42, 60, and 70 days of age receiving; Figure S2. White blood cell differential count metabolites in calves at 7, 21, 42, 60, and 70 days of age; Figure S3. Rumen fluid pH, total VFA concentration, and molar proportions of acetic acid, propionic acid, butyric acid, isobutyric acid, valeric acid and isovaleric acid in calves at 60 d and 70 d of age; Figure S4. Rumen fluid acetic acid/propionic acid ratio, acetic acid plus butyric acid/propionic acid ratio, ammonia-N, D- and L-lactate in calves at 60 d and 70 d of age.

## Abbreviations

The following abbreviations are used in this manuscript:

(C2 + C3)/C4	sum of acetic acid and propionic acid to butyric acid
ADG	average daily gain
AI	artificial insemination
AICC	Akaike information criterion corrected
ALP	alkaline phosphatase
AOPP	advanced oxidation protein products
AST/GOT	aspartate-aminotransferase
BHB	beta-hydroxybutyrate
BW	body weight
CTR	control group
C2/C3	ratios of acetic acid to propionic acid
DIM	days in milk
DM	dry matter
DMI	dry matter intake
ECM	energy corrected milk
EDTA	ethylenediaminetetraacetic acid
FRAP	ferric reducing antioxidant power
GGT	γ-glutamyl transferase
GPFT	genomic productivity, functionality and type index
HG	heart girth
LPS	lipopolysaccharide
LSM	least squares means
MFI	mean fluorescent intensity
MPO	myeloperoxidase
MR	milk replacer
NEFA	nonesterified fatty acids
PBS	phosphate-buffered saline
PMN	polymorphonuclear neutrophils
PON	paraoxonase
qPCR	quantitative PCR
ROM	total reactive oxygen metabolites
SCFP	*Saccharomyces cerevisiae* fermentation-derived postbiotic
TMR	total mixed ration
TRT	treatment
VFA	volatile fatty acids
WBA	whole blood stimulation assay
WBC	total white blood cells
WH	wither height
