# Supplementary material for: Saccharomyces cerevisiae Fermentation-Derived Postbiotics Supplementation to Dairy Calves: Effects on Growth, Metabolism, Immune Status and Preliminary First Lactation Outcomes
Source: Animals (Basel). 2025 Sep 18;15(18):2728. doi: 10.3390/ani15182728 (PMC12466413; doi:10.3390/ani15182728)
Supplement: Supplementary file 1 [file animals-15-02728-s001.zip › animals-3853365-supplementary.pdf]

# SUPPLEMENTAL FILE

***Gas chromatography for VFA analysis***

The concentration of VFA was analyzed by gas chromatography using a gas chromatograph (model 7820A; Agilent Technologies, Santa Clara, CA) equipped with a capillary column (30 m × 250 µm × 0.25 µm; DB-FFAP, Agilent J&W GC column) and a flame ionization detector according to Ahmed et al., 2013. The oven temperature was maintained at 70°C for 3 minutes and then increased by 8°C/min to 150°C, then increased by 20°C/min to 190°C and maintained for 3 minutes. The injector temperature was 250°C and the detector temperature was 300°C. The inlet was equipped with a glass wool lining to separate dirt particles from the sample. Samples were injected using an automatic sampler at an injection size of 1 µL using the split method and a 25:1 splitting ratio for rumen and fecal samples and a 10:1 splitting ratio for plasma samples. Hydrogen and air were used for flame ionization detection. The carrier gas was nitrogen, with a constant flow rate of 1.78 mL/min. Individual volatile fatty acids were identified based on their retention time and the results compared to a standard blend of fatty acids of known concentration. The concentration of the single volatile fatty acids in the sample was determined from the ratio of the areas of the single fatty acids in the standard mixture.

Data from the rumen and fecal samples were expressed as concentration for the total amount of VFA and as the molar percentage of each VFA relative to the total VFA. The ratios of acetic acid to propionic acid (C2/C3) and the sum of acetic acid and propionic acid to butyric acid ((C2+C3)/C4) were calculated. For plasma, data of the total amount of VFA and the individual VFAs were expressed as concentrations

**Supplemental Table S1.** GenBank accession number, hybridization position, sequence, amplicon size and source of primers for Bos taurus used to analyze gene expression by qPCR.

| Accession no. | Gene | Primers[1] | Primers (5'-3') | (bp)[2] |
|---|---|---|---|---|
| XM_015459671.1 | *CCR2* | F.138 | CGTGGGACAAATCGAAGCAC | 199 |
| | | R.336 | AGCATAGTGAGCCCAGAACG | |
| NM_001102558.2 | *CX3CR1* | F.75 | CCCAGCCCAGGTGCTCA | 175 |
| | | R.249 | CAGCAAATTTCCCACCAGGC | |
| NM_198221.2 | *ITGAL* | F.1582 | ATCAACGGGGATGAGCTGAC | 126 |
| | | R.1707 | GGTCCCTTCTATCCGCTGAC | |
| NM_175781.1 | *ITGB2* | F.1231 | GACACCCTGAAAGTCACCTACGA | 108 |
| | | R.1338 | GAAGGTGATCGGGACGTTGAT | |
| NM_174744.2 | *MMP9* | F.1169 | GCCCGGATCAAGGATACAGC | 128 |
| | | R.1296 | GGGGTGCTCCTCTGTGAATC | |
| NM_174182.1 | *SELL* | F.588 | CTCTGCTACACAGCTTCTTGTAAACC | 104 |
| | | R.691 | CCGTAGTACCCCAAATCACAGTT | |
| NM_001037628.2 | *SELPLG* | F.123 | CTGAGCACGGTGCCATGTTTC | 111 |
| | | R.233 | CTGGGGCCTTCACAGTTTCA | |
| NM_174008.1 | *CD14* | F.525 | TCCGTAACGTATCGTGGACAAC | 100 |
| | | R.624 | GAGTGTGCTTGGGCAATGTTC | |
| XM_003586675.4 | *LCN2* | F.749 | CCAGTGAGCCTGCACCTTTG | 141 |
| | | R.889 | TATTTAGCAGGCAAGGCAGGG | |
| NM_001113298.2 | *MPO* | F.1311 | AGCCATGGTCCAGATCATCAC | 105 |
| | | R.1415 | ACCGAGTCGTTGTAGGAGCAGTA | |
| NM_174615.2 | *SOD1* | F.256 | GGCTGTACCAGTGCAGGTCC | 101 |
| | | R.356 | GCTGTCACATTGCCCAGGT | |
| NM_201527.2 | *SOD2* | F.620 | TGTGGGAGCATGCTTATTACCTT | 95 |
| | | R.714 | TGCAGTTACATTCTCCCAGTTGA | |
| NM_174197.2 | *TLR2* | F.3182 | CCATGTCTGGAGAGGGTGTT | 102 |
| | | R.3283 | GGGGACACAAAACAGCACTT | |
| NM_174178.2 | *SDHA* | F.1563 | CTGAAGCAGGTTTCAACACG | 130 |
| | | R.1544 | GTTGTCCTCCTCCATGTTCC | |
| NM_173925.2 | *IL8* | F.350 | GTGAAGAGAGCTGAGAAGCAAG | 150 |
| | | R.499 | CACCAGACCCACACAGAACAT | |
| NM_001034034.2 | *GAPDH* | F.276 | GAAGCTCGTCATCAATGGAAA | 60 |
| | | R.335 | CCACTTGATGTTGGCAGGAT | |
| NM_001076998.2 | *RPL13A* | F.417 | CTCGCAAGTTTGCCTACCTA | 113 |
| | | R.529 | CCGATAGTGGATCTTGGCCT | |
| NM_001075742.1 | *TBP* | F.366 | GAAGGTGAACGTCATGGATCAG | 118 |
| | | R.483 | GCCGTAAGGCATCATTGGA | |
| NM_001278621.1 | *CD36* | F.743 | GTACAGATGCAGCCTCATTTCC | 81 |
| | | R.823 | TGGACCTGCAAATATCAGAGGA | |
| NM_001030301.1 | *TICAM1* | F.1013 | AAGACACCGCTTACCTGTCG | 113 |
| | | R.1125 | AGGGGTCGATGGAGAAGGAA | |
| NM_174198.6 | *TLR4* | F.555 | TGCGTACAGGTTGTTCCTAACATT | 110 |
| | | R.664 | TAGTTAAAGCTCAGGTCCAGCATCT | |
| NM_001078159.1 | *LYZ* | F.281 | AAAGCAGTTAACGCCTGTCGTAT | 122 |
| | | R.402 | CATGCCACCCATGCTTTAATG | |
| NM_180998.2 | *LTF* | F.631 | CCAGGGAGCTGTGGCTAAAT | 135 |
| | | R.765 | AAGTATGGTTCCCGGGAGGA | |

**Supplemental Table S1.** GenBank accession number, hybridization position, sequence, amplicon size and source of primers for Bos taurus used to analyze gene expression by qPCR.

| Accession no. | Gene | Primers[1] | Primers (5'-3') | (bp)[2] |
|---|---|---|---|---|
| NM_001076799.1 | *NOS2* | F.1690 | GCCTTCAACCCCAAGGTTCT | 102 |
| | | R.1791 | GTCTCCGTTGCCAAAAGTGC | |
| NM_001102219.1 | *NLRP3* | F.69 | CTTTCTGGACTCTGACCGGG | 149 |
| | | R.217 | ATTGAGGTGCAGCCCTTCTG | |
| NM_001040555.1 | *IRAK1* | F.950 | CCTCAGCGACTGGACATCCT | 103 |
| | | R.1052 | GGACGTTGGAACTCTTGACATCT | |
| NM_008361.4 | *IL1B* | F.120 | GCCACCTTTTGACAGTGATGAG | 137 |
| | | R.256 | TGATGTGCTGCTGCGAGATT | |
| NM_001206735.1 | *IL1R* | F.897 | CCCGGGCGATAAAGCTGATT | 104 |
| | | R.1000 | AAGCCAGGATCCCAAGACCA | |
| NM_173921.2 | *IL4* | F.239 | CTGCCCCAAAGAACACAACTG | 106 |
| | | R.344 | AGTCCGCCCAGGAATTTGTT | |

[1] Primer direction (F – forward; R – reverse) and hybridization position on the sequence. are underlined.
[2] Amplicon size in base pair (bp).

**Supplemental Table S2.** Arbitrary mRNA abundance for gene expression in blood leucocytes after the ex-vivo whole blood stimulation assay with Lipopolysaccharides (LPS) in calves at 60 and 70 days of age receiving either no supplementation (CTR; n = 9) or 1 g/d of SmartCare® (Diamond V™) in the milk replacer from 3 to 60 d along with 5 g/d of NutriTek® (Diamond V™) from 3 to 70 d (SCFP; n = 9).

| | **TRT** | | | ***P*-value** | | |
|---|---|---|---|---|---|---|
| **Gene** | CTR | SCFP | SEM | TIME | TRT | TIMExTRT |
| ***Recognition and immune mediation functions*** | | | | | | |
| *CD14* | 2524.77 | 2505.28 | 243.69 | 0.43 | 0.95 | 0.15 |
| *CD36* | 26.09 | 24.49 | 3.72 | <0.01* | 0.69 | 0.21 |
| *TLR4* | 293.73 | 271.68 | 28.14 | 0.67 | 0.48 | 0.55 |
| *TLR2* | 526.02 | 502.26 | 34 | 0.03* | 0.59 | 0.18 |
| ***Migration and cell adhesion*** | | | | | | |
| *ITGB2* | 364.93 | 390.08 | 23.08 | 0.78 | 0.24 | 0.45 |
| *ITGAL* | 165.35 | 173.15 | 10.53 | 0.03* | 0.53 | 0.17 |
| *SELL* | 606.22 | 638.23 | 46.21 | 0.62 | 0.45 | 0.72 |
| *SELPLG* | 537.72 | 564.07 | 23.59 | 0.69 | 0.21 | 0.87 |
| ***Antimicrobial strategies*** | | | | | | |
| *MMP9* | 5.72 | 5.69 | 0.78 | 0.01* | 0.97 | 0.72 |
| ***Oxidative stress*** | | | | | | |
| *SOD1* | 602.58 | 572.17 | 41.09 | 0.54 | 0.57 | 0.73 |
| *NOS2* | 384.71 | 318.67 | 48.08 | 0.38 | 0.3 | 0.27 |

Data are presented as mean and SEM. Significance levels of the main effects of the models are reported.

* Indicates a significant difference (P ≤ 0.05), + Indicates a trend (P < 0.1).

TRT = treatment.

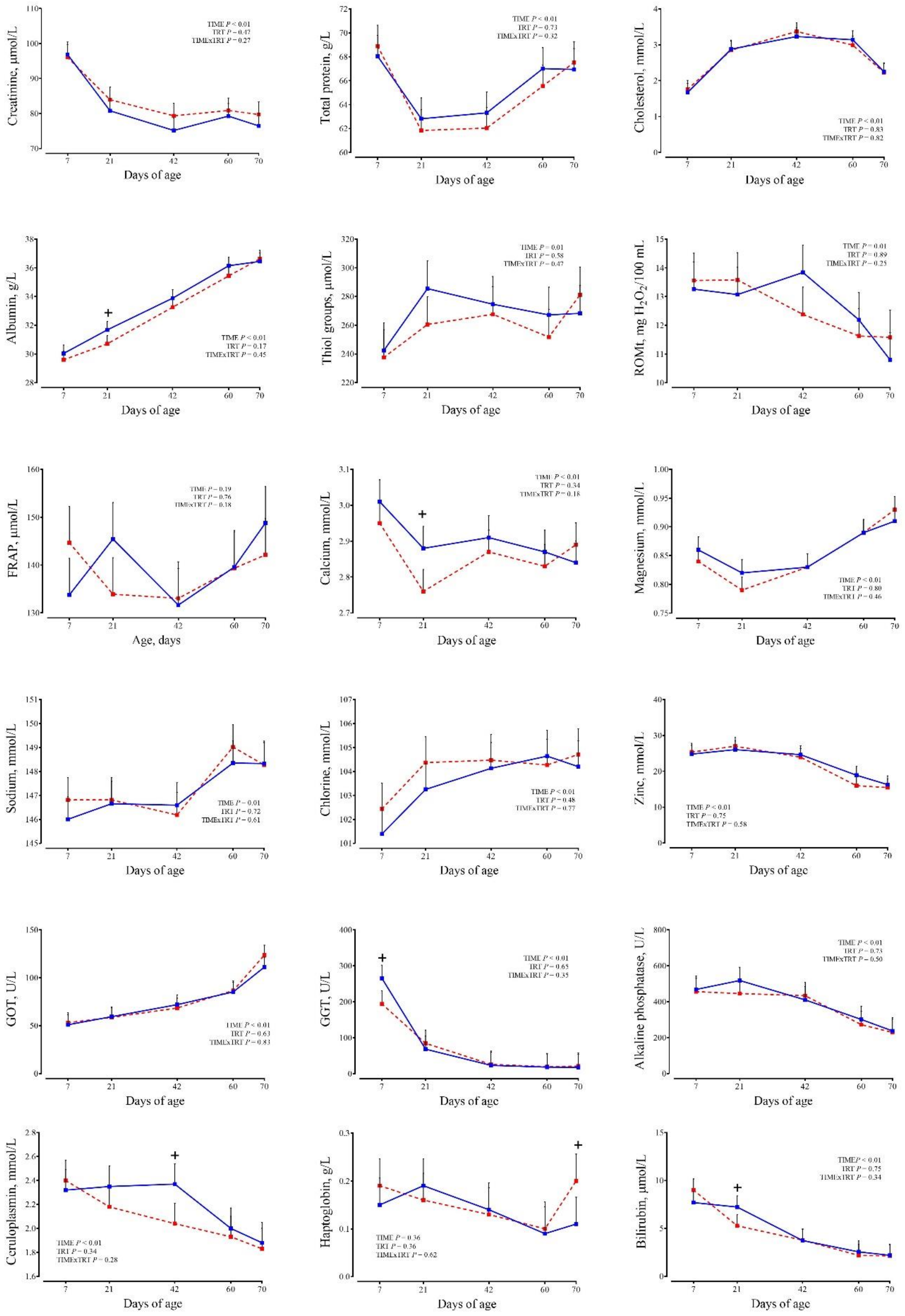


**Supplementary figure S1**. Concentrations of plasma metabolites in calves at 7, 21, 42, 60, and 70 days of age receiving either no supplementation (CTR, blue circles and solid lines; n = 9) or 1 g/d of SmartCare® (Diamond V™) in the milk replacer from 3 to 60 d along with 5 g/d of NutriTek® (Diamond V™) from 3 to 70 d (SCFP, red squares and dashed lines; n = 9). Data are presented as mean and SEM. Significance levels of the main effects of the models are reported. * Indicates a significant difference ($P \leq 0.05$), + Indicates a trend ($P < 0.1$). TRT = treatment.

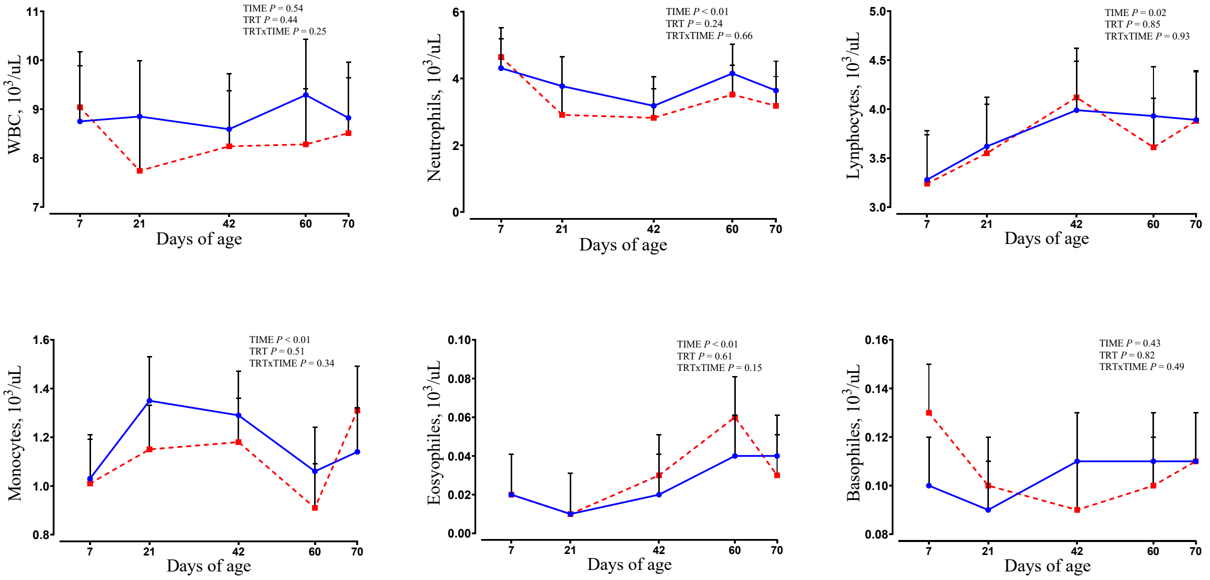


**Supplementary figure S2**. White blood cell differential count metabolites in calves at 7, 21, 42, 60, and 70 days of age receiving either no supplementation (CTR, blue circles and solid lines; n = 9) or 1 g/d of SmartCare® (Diamond V™) in the milk replacer from 3 to 60 d along with 5 g/d of NutriTek® (Diamond V™) from 3 to 70 d (SCFP, red square and dashed lines; n = 9). Data are presented as mean and SEM. Significance levels of the main effects of the models are reported. * Indicates a significant difference ($P \leq 0.05$), + Indicates a trend ($P < 0.1$). TRT = treatment.

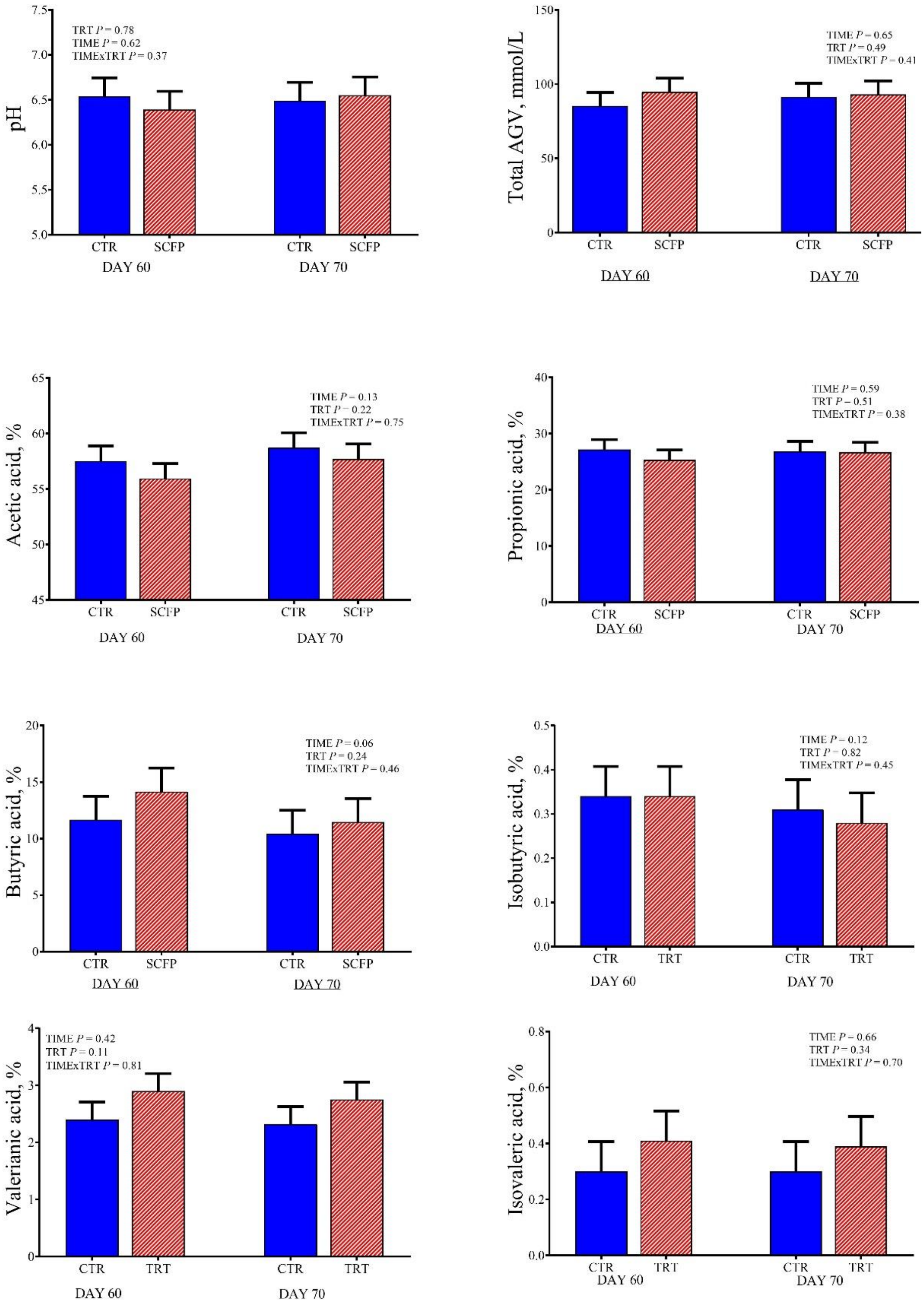


**Figure S3**. Rumen fluid pH, total VFA concentration, and molar proportions of acetic acid, propionic acid, butyric acid, isobutyric acid, valeric acid and isovaleric acid in calves at 60 d and 70 d of age receiving either no supplementation (CTR, blue full columns; n = 9) or 1 g/d of SmartCare® (Diamond V™) in the milk replacer from 3 to 60 d along with 5 g/d of NutriTek® (Diamond V™) from 3 to 70 d (SCFP, red striped columns; n = 9). Data are presented as mean and SEM. Significance levels of the main effects of the models are reported. * Indicates a significant difference ($P \leq 0.05$), + Indicates a trend ($P < 0.1$). TRT = treatment.

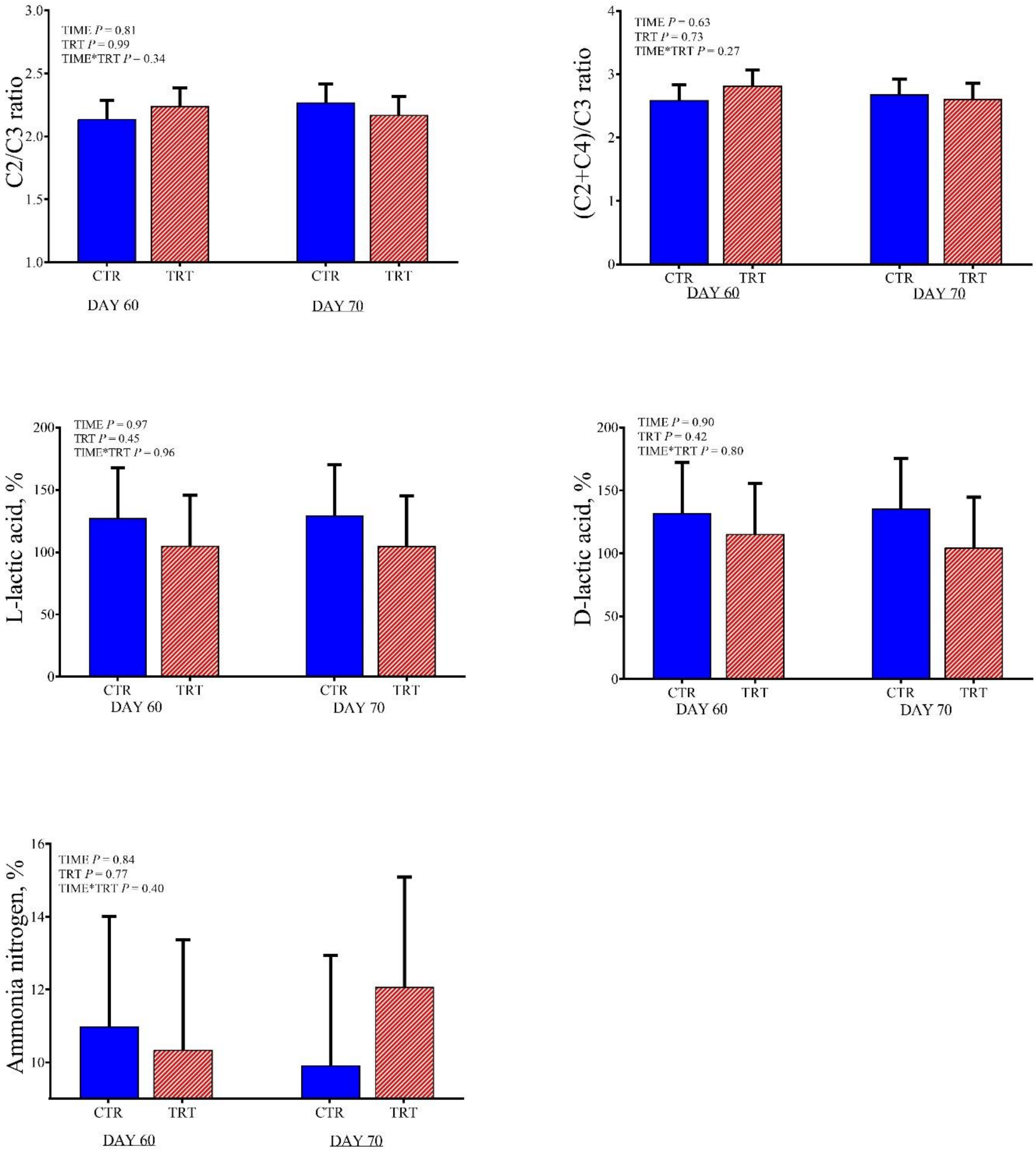


**Figure S4**. Rumen fluid acetic acid/ propionic acid ratio, acetic acid plus butyric acid/ propionic acid ratio, ammonia-N, D- and L-lactate in calves at 60 d and 70 d of age receiving either no supplementation (CTR, blue full columns; n = 9) or 1 g/d of SmartCare® (Diamond V™) in the milk replacer from 3 to 60 d along with 5 g/d of NutriTek® (Diamond V™) from 3 to 70 d (SCFP, red striped columns; n = 9). Data are presented as mean and SEM. Significance levels of the main effects of the models are reported. * Indicates a significant difference ($P \leq 0.05$), + Indicates a trend ($P < 0.1$). TRT = treatment.
